# Collaborative Metabolism: Gut Microbes Play a Key Role in Canine and Feline Bile Acid Metabolism

**DOI:** 10.3390/vetsci11020094

**Published:** 2024-02-18

**Authors:** John C. Rowe, Jenessa A. Winston

**Affiliations:** 1 Department of Veterinary Clinical Sciences, The Ohio State University College of Veterinary Medicine, Columbus, OH 43210, USA; rowe.285@buckeyemail.osu.edu; 2 Comparative Hepatobiliary Intestinal Research Program (CHIRP), The Ohio State University College of Veterinary Medicine, Columbus, OH 43210, USA

**Keywords:** bile acids, gut microbiota, bile acid biotransformation, bai operon, feline, canine, microbial-derived bile acids, secondary bile acids, BSH, bile salt hydrolase

## Abstract

**Simple Summary:**

This review explores the emergence of new literature connecting microbial-derived bile acid metabolism to canine and feline health and disease. Specifically, it highlights how gut microbes can orchestrate canine and feline physiology and disease through metabolism and the diversification of bile acid pools, which ultimately impact the host via the activation of bile acid receptors. Incorporating the therapeutic potential of bile acid metabolism into evidence-based veterinary practice will require familiarity with and an understanding of these concepts for a broad array of veterinarians including general practitioners, specialists, and researchers.

**Abstract:**

Bile acids, produced by the liver and secreted into the gastrointestinal tract, are dynamic molecules capable of impacting the overall health of dogs and cats in many contexts. Importantly, the gut microbiota metabolizes host primary bile acids into chemically distinct secondary bile acids. This review explores the emergence of new literature connecting microbial-derived bile acid metabolism to canine and feline health and disease. Moreover, this review highlights multi-omic methodologies for translational research as an area for continued growth in veterinary medicine aimed at accelerating microbiome science and medicine as it pertains to bile acid metabolism in dogs and cats.

## 1. Introduction

The microbial community within the gastrointestinal tract is vast and contains billions of metabolically active microbes. The metabolic products both from host cells and microbes can act as signaling molecules and impact host physiology. In recent years, bile acids (BAs) are one of these metabolites that have been increasingly described in dogs and cats. Both the host and gut microbes collaboratively biotransform BAs into metabolites that can be sensed by the host and directly impact states of health and disease.

This review aims to address three areas pivotal for understanding the physiologic role of microbial-derived BAs in dogs and cats: (1) the physiology of BAs and the ability of the gut microbes to diversify the BA pools; (2) a literature review of microbial-derived BAs in dogs in health and disease; (3) a literature review of microbial-derived BAs in cats in health and disease. This topic has not been reviewed previously in the veterinary literature. Throughout this review, the translational application of microbiome science is emphasized, as new discoveries drive the understanding of health and treatment of disease in both veterinary and human patients.

## 2. Collaborative Physiology of Bile Acid Metabolism

### 2.1. Bile Acids as Signaling Molecules—Moving beyond Digestion

**Figure 1:**
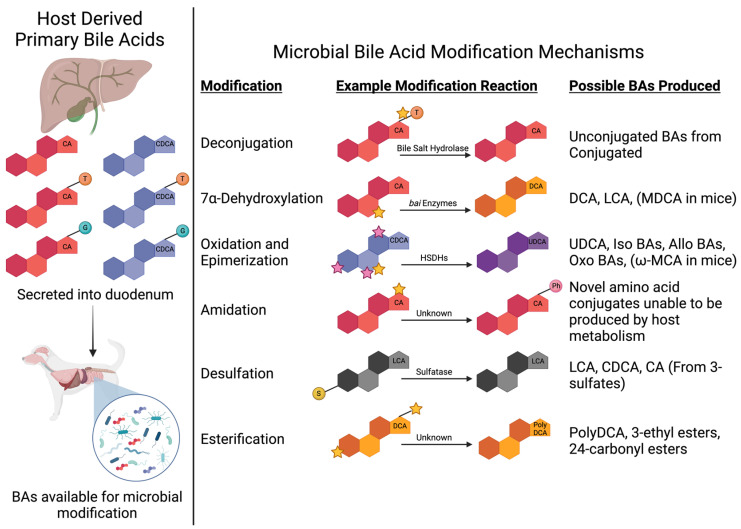
Bile acid transformation. Host-derived primary bile acids are depicted in the left panel in either a conjugated or unconjugated state, where they are then secreted into the intestinal tract and can be available for microbial modification. Conjugation is represented by a colored sphere (blue for glycine and orange for taurine) attached to the major ringed hydrocarbon molecule bile acid structure. The right panel depicts reactions performed by gut microbes that produce microbial-derived secondary bile acids. Stars depict the molecular locations where the listed reactions occur. Figure was created with BioRender.com. Abbreviations: BA = bile acid; BSH = bile salt hydrolase; HSDH = hydroxysteroid dehydrogenase; T = taurine; G = glycine. Primary bile acid abbreviations used: CA = cholic acid, CDCA = chenodeoxycholic acid. Secondary bile acid abbreviations used: DCA = deoxycholic acid; LCA = lithocholic acid; UDCA = ursodeoxycholic acid; ωMCA = omega muricholic acid. Amino acid abbreviations used: Ph = phenylalanine.

Bile acids are lipid molecules synthesized in the liver and secreted into the intestinal tract via the biliary system. Aiding in the digestion and absorption of fat is a core function of BAs. This function requires that BAs have amphipathic molecular properties, which promote lipid and water solubility at different molecular locations. Primary bile acids (PBAs) are derived from cholesterol, which has an inherent four steroid ring structure. The ringed portion of BA molecular structure is hydrophobic and promotes lipid solubility (Figure 1). Meanwhile, hydroxyl groups positioned around the ring structure provide a polar component that is hydrophilic and promotes water solubility. Further, the addition of the amino acids taurine or glycine to the side chain of the BA, termed conjugation, provides an additional hydrophilic region to the molecule that promotes water solubility. Functionally, this permits hydrophobic regions to interact with ingested fats, while hydrophilic regions allow the BAs to be dissolved in aqueous solutions, including blood. When multiple BAs and fat molecules form this assembly, it is termed a micelle and is crucial for dietary fat absorption [1]. Moreover, the mix of hydrophobic and hydrophilic properties also allow BAs to function as detergents and disrupt cell membranes, including those of microbes [2]. The classic roles BAs play in digestion and as detergents remain integral to their biology but are only the beginning of their vast physiologic functions.

Aside from the physical molecular roles BAs play in digestion and as detergents, BAs serve as signaling molecules [3,4]. Sensed by a variety of host receptors, which will be discussed in more detail within this review, BAs modulate host physiology ranging across host metabolism, immune function, and cell survival [3]. In health, rodent models and human clinical studies have demonstrated key roles for BAs regulating glucose and lipid metabolism [5,6], insulin sensitivity [7], inflammation and immunity [8,9], and thyroid-mediated energy expenditure [10]. Further roles in disease states including *Clostridioides difficile* infection [11], inflammatory bowel disease (IBD) [12,13,14], neurologic disease [15], cardiovascular disease [16], as well as obesity, type 2 diabetes, dyslipidemia, and nonalcoholic fatty liver disease [17] have also been characterized. With so many emerging applications for BA signaling to impact host physiology, over the past 15 years, research has been focused on defining collaborative BA metabolism, BA signaling, and factors that can modulate both. 

Microbes within the gastrointestinal tract, known as the gut microbiome, modify host-derived BAs into secondary bile acids (SBAs) [18]. These microbial biotransformations and modifications fundamentally alter the signaling potential of BAs [19,20]. This highlights the importance of the gut microbiome and its functional potential to impact host physiology through the diversification of BA pools. Therefore, it is crucial to understand BA metabolism as a product of collaborative metabolism by the host and gut microbes. 

Increasingly in veterinary medicine, the canine and feline gut microbiome has been characterized in health and states of disease [21,22]. Similar to human medicine, there is a growing body of evidence that BAs impact veterinary patients in health and disease, which this review addresses and summarizes for the first time. Prior to exploring the current veterinary literature, the next section will review collaborative BA metabolism between the host and gut microbiome and illustrate the physiologic impact of BAs in canine and feline health and disease.

### 2.2. Microbial Modifications Expand Bile Acid Pool Diversity and Signaling Potential

Primary bile acids, cholic acid (CA) and chenodeoxycholic acid (CDCA), are synthesized from cholesterol by hepatocytes [23]. Bears and mice are capable of producing ursodeoxycholic acid (UDCA) as a PBA [24,25]. Mice have the additional capability to produce α-muricholic acid (αMCA) and β-muricholic acid (β-MCA) as PBAs [25,26]. Dogs, cats, and humans are only capable of producing the two PBAs, CA and CDCA. These host-derived PBAs are commonly conjugated with the amino acids, specifically taurine and glycine, prior to entering the biliary tract [27]. Since both CA and CDCA can be unconjugated or conjugated to taurine or glycine, this results in six possible host-derived PBAs released into the duodenum by dogs and cats. Cats almost exclusively conjugate BAs with taurine (98.4% taurine conjugated BAs, 1.1% glycine conjugated BAs, and 0.5% unconjugated BAs) [28]. Cats suffering from taurine deficiency have a significant overall decrease in BA production and secretion capability [28,29]. In dogs, there is a similar bias with taurine BA conjugation, making up at least 99% of total BAs within bile [30,31,32]. The bias for taurine BA conjugation seen in dogs and cats is not observed in humans, where, conversely, it is well established that glycine BA conjugation predominates [33,34]. Functionally, conjugated BAs exhibit amphipathic behavior, allowing the emulsification of dietary lipids and promoting lipid and fat-soluble vitamin absorption [35].

**Figure 2:**
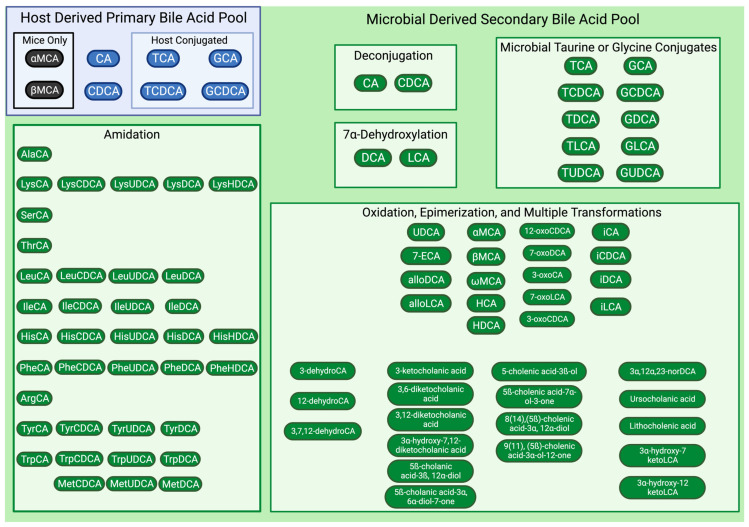
Microbial diversification of host primary bile acids. Host-derived primary bile acids are shown within the light blue box with primary bile acids of dogs, cats, and humans depicted as blue ovals and with additional primary bile acids of rodents depicted as black ovals. Reactions performed by gut microbes that produce secondary bile acids title each light green box. Resulting microbial-derived secondary bile acids are depicted as green ovals. Though not comprehensive, the shown microbial-derived secondary bile acids represent those described by Quinn et al., 2020, Guzior and Quinn, 2021, and Zhu et al., 2022, as well as bile acids that can be assessed using the metabolomics fee-for-service laboratory at Pennsylvania State University. Figure was created with BioRender.com. Primary bile acid abbreviations used: CA = cholic acid, CDCA = chenodeoxycholic acid, GCA = glycocholic acid, GCDCA = glycochenodeoxycholic acid, TCA = taurocholic acid, TCDCA = taurochenodeoxycholic acid, αMCA = alpha muricholic acid, βMCA = beta muricholic acid. Secondary bile acid abbreviations used: DCA = deoxycholic acid, ECA = epicholic acid, GDCA = glycodeoxycholic acid, GLCA = glycolithocholic acid, GUDCA = glycoursodeoxycholic acid, HCA = hyocholic acid HDCA = hyodeoxycholic acid, LCA = lithocholic acid, TDCA = taurodeoxycholic acid, TLCA = taurolithocholic acid, TUDCA = tauroursodeoxycholic acid, UCA = ursocholic acid, UDCA = ursodeoxycholic acid, γMCA = gamma muricholic acid, ωMCA = omega muricholic acid. Amino acid abbreviations used: Ala = alanine, Lys = lysine, Ser = serine, Thr = threonine, Leu = leucine, Ile = isoleucine, His = histidine, Phe = phenylalanine, Arg = arginine, Tyr = tyrosine, Trp = tryptophan, Met = methionine.

Once BAs enter the intestinal tract, microbial diversification of the BA pool from the original six host-derived PBAs begins (Figure 2). The deconjugation of conjugated PBAs is the first step of BA modification mediated by gut microbes and occurs primarily in the distal small intestine and colon [36,37]. Gut microbes utilize bile salt hydrolases (BSHs) to remove the taurine or glycine from conjugated PBAs, therefore returning the BA to an unconjugated state which is required for reabsorption [37]. Major genera that contribute to BSH activity include *Clostridium*, *Lactobacillus*, *Bifidobacterium*, *Bacteroides*, and *Enterococcus*, with crystal structures identified from four BSHs generated by *Clostridium perfringens*, *Lactobacillus salivarius*, *Bifidobacterium longum*, and *Enterococcus faecalis* [18,38]. As signaling molecules, conjugated BAs display varying affinity for host BA-activated receptors such as the farnesoid x receptor (FXR) and Takeda G protein-coupled receptor 5 (TGR5). Therefore, microbially mediated BA deconjugation diversifies the overall BA pool, thus broadening the signaling potential of host BA [38]. Additionally, deconjugation releases taurine and glycine to be utilized in microbial metabolism and reduces the inherent detergent activity of conjugated BAs [18]. 

Beyond BSH activity, it has recently been described that gut microbes are capable of creating novel amino acid conjugates of host BAs with phenylalanine, leucine, tyrosine, proline, and alanine [39,40]. Moreover, in human and mouse feces, as many as 118 novel amino acid-conjugated bile acids, also known as microbial conjugated BAs, have been recently identified [41]. The process of amino acid addition to an existing unconjugated BA is termed amidation (Figure 1). Importantly, these newly discovered microbial-derived BA–amino acid conjugates demonstrate affinity for host BA-activated receptors, including FXR [39], highlighting the need to consider their role in regulating host physiology. When phenylalanine, leucine, and tyrosine BA conjugates were first described, an analysis of existing publicly available mass spectrometry data found that these novel microbial conjugated BAs were enriched in human infants as well as humans with IBD and cystic fibrosis [39]. At this time, there are no known mammalian metabolic pathways that yield these novel microbial conjugated BAs, and these novel BA conjugates do not appear susceptible to BSH deconjugation [39]. To date, these microbial conjugated BAs are not described in dogs and cats. However, in the authors’ opinion, novel amino acid-conjugated BAs likely exist in dogs and cats, and as described in humans, they likely contribute to host physiology and may impact disease pathogenesis.

In addition to manipulating the status of BA conjugation through deconjugation and amidation, gut microbes convert host unconjugated PBAs into secondary bile acids (SBAs). The production of microbial-derived SBAs greatly expands the diversity and functionality of the BA pools. While it has been reviewed previously [18], BA diversification by the gut microbiota represents an integral component of dynamic physiology where microbial metabolites allow communication and signaling between the host and the gut microbiome. The chemical modification of host-derived PBAs into microbial-derived SBAs relies upon key BA biotransformations, specifically dehydroxylation, oxidation or reduction, and epimerization [42]. To a lesser extent, the microbial esterification of BAs also contributes to diversification, but currently, the microbial enzymes performing esterification are unknown [43]. Lastly, while reviewed elsewhere [44], a barrier to microbial BA biotransformation is the addition of a sulfate group to the BA by host sulfatase enzymes, termed sulfation. Sulfated BAs cannot undergo microbial biotransformation [44]. Gut microbes possess the ability to overcome this barrier through desulfation, a process that removes the sulfate group [45]. Collectively, the mechanisms behind microbial-derived SBA generation highlights the importance of a diverse gut microbiome capable of performing these chemical modifications.

The largest driver in generating microbial-derived SBAs is the multistep process of 7α-dehydroxylation (Figure 1 and Figure 2) where deoxycholic acid (DCA) is created from CA and lithocholic acid (LCA) is created from CDCA [18,42]. These biotransformation processes occur in a sequence of eight steps from genes encoded by the bile acid-inducible (*bai*) operon [46]. This function is carried out by a select few gut microbes, which in humans accounts for less than 0.025% of the colonic microbiota [47]; however, this information is unknown in dogs and cats. The main organisms identified in humans to perform 7α-dehydroxylation are *Clostridium scindens*, *Clostridium hylemonae*, *Clostridium sordelli*, and *Peptacetobacter* (formerly *Clostridium*) *hiranonis* [18,42]. In mice, *Extibacter muris* is also capable of producing DCA and LCA [48]. Further, recent evidence from the metagenomic sequencing of human stool has identified a cluster of uncultured bacteria affiliated with the family *Oscillospiraceae* where 41 metagenome-assembled genomes (MAGs) containing the full complement of *bai* genes were identified including novel members within the *Ruminococcaceae* and *Lachnospiraceae* families [49,50]. 

Within veterinary medicine, much attention has been rightly devoted to the 7α-dehydroxylation ability of *Peptacetobacter hiranonis* (formerly *Clostridium hiranonis*). This organism is considered a key feature of the normal, healthy gut microbiome of dogs [22]. A full genome from *P. hiranonis* has been characterized from a canine fecal isolate [51] and the complete *bai* operon identified, confirming the genetic capability to perform 7α-dehydroxylation [52]. Further, the absence of *P. hiranonis* from the gut microbiome is an indicator associated with dysbiotic states in dogs [53] and cats [54]. Moreover, despite the evidence in humans and mice demonstrating multiple microbes capable of 7α-dehydroxylation, to the authors’ knowledge, there are no other 7α-dehydroxylating bacteria that have been isolated or shown to be present in the gut microbiota of dogs or cats. 

Following 7α-dehydroxylation, further diversification of the BA pools is achieved via microbial oxidation, reduction, and epimerization reactions (Figure 1 and Figure 2). These microbial reactions can also create SBAs from host-derived PBAs. For example, the conversion of CDCA to ursodeoxycholic acid (UDCA) occurs through oxidation and subsequent epimerization reactions via 7α- and 7β-hydroxysteroid dehydrogenase [18,42]. UDCA is commercially available as Ursodiol and is thus therapeutically utilized in both human and veterinary medicine, including approaches that capitalize on the anti-inflammatory properties UDCA mediates through host BA-activated receptors [55,56]. In humans, host PBAs undergo oxidations, reductions and epimerizations, typically at the third, sixth, seventh, and twelfth carbons, resulting in the microbial production of over 50 chemically distinct SBAs [18,20,57]. The microbes known to perform these functions include members of the phyla Actinobacteria (newly named Actinomycetota), Proteobacteria (newly named Pseudomonadota), Firmicutes (newly named Bacillota), and Bacteroidetes (newly named Bacteroidota) [20]. These reactions can also occur to change the structure of one microbial-derived SBA into another. With each change in chemical structure, there is a corresponding change to the functional potential of the BA. In health, by the time feces is expelled, the BA composition is dominated by microbial-derived SBAs [58,59].

### 2.3. Bile Acid Reabsorption and Host Receptor Affinity

The interactions of BAs with host cellular proteins facilitate both BA reabsorption and subsequent molecular signaling. Remarkably, 95% of BAs initially secreted into the intestinal lumen undergo enterohepatic recirculation and are retrieved from the intestinal lumen, with only the remaining 5% entering the colon [60]. A minor contribution of the passive reabsorption of BAs occurs within the jejunum and colon [60,61,62]. The majority of BA reabsorption is the result of active transport in the ileum mediated by an apical sodium-dependent bile acid transporter (ASBT), which cotransports BAs with sodium ions and is well described in humans [35,60,61,63]. Briefly, ASBT is primarily expressed on the ileal brush border but can be found at a lower density in the duodenum and jejunum of humans and is thought to be restricted to ileal expression in rats, mice, hamsters, and monkeys [61]. Recently, in healthy dogs (*n* = 11), immunohistochemistry revealed that ASBT is highly expressed in ileal villous enterocytes but is absent in the crypts [64]. ASBT is expressed to a lesser extent multifocally on superficial enterocytes of the canine cecum and colon [64]. In situ hybridization also demonstrated that ASBT mRNA is minimally expressed in the canine duodenum and jejunum, though the ASBT protein was not detected in these locations [64]. Interestingly, dogs with chronic inflammatory enteropathy (*n* = 24) expressed significantly less ASBT in the ileum, and ileal ASBT expression negatively correlated with the increasing severity of inflammatory histopathologic scores [64]. Reduced ASBT expression is also described in people suffering from Crohn’s disease, a form of IBD [65]. Interesting, ASBT expression improves in patients with Crohn’s disease treated with glucocorticoids; to the authors’ knowledge, this has not been evaluated in dogs [65]. Of note, intestinal ASBT expression in cats has yet to be characterized. 

**Figure 3:**
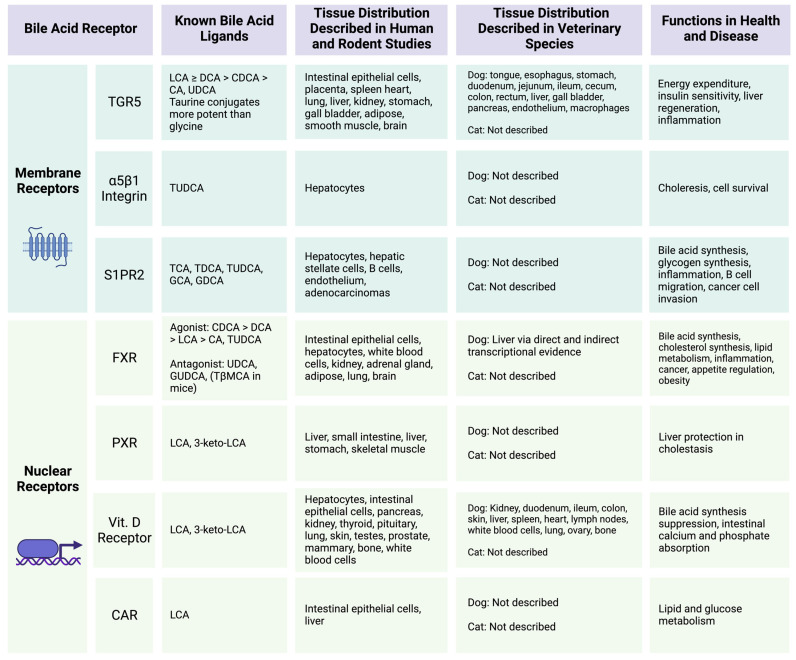
Host bile acid-activated receptors and functions. Summary of known host receptors that respond to bile acid ligands. Figure was created with BioRender.com. References corresponding to information within table: TGR5 [66,95,96,97,98,99,106,107,108,109,110,111,112], a5b1 [100], S1PR2 [66,101,102,103,104,105], FXR [66,67,68,69,70,71,72,73,74,75,85,86,87,88,91,92], PXR [66,81,82,83], Vitamin D Receptor [66,76,77,78,79,80], CAR [66,84]. Primary bile acid abbreviations used: CA = cholic acid, CDCA = chenodeoxycholic acid, GCA = glycocholic acid, TCA = taurocholic acid, GCDCA = glycochenodeoxycholic acid, TβMCA = tauro beta muricholic acid. Secondary bile acid abbreviations used: DCA = deoxycholic acid, GDCA = glycodeoxycholic acid, GUDCA = glycoursodeoxycholic acid, LCA = lithocholic acid, TDCA = taurodeoxycholic acid, TLCA = taurolithocholic acid, TUDCA = tauroursodeoxycholic acid, UDCA = ursodeoxycholic acid.

Broadly, BA signaling is mediated by two types of host receptors: nuclear and membrane-bound (Figure 3). The best characterized BA-activated nuclear receptor is FXR [66,67,68,69,70,71,72,73]. Bile acids that bind FXR do so with varied affinity such that the strength of the binding affinity follows the order of CDCA > DCA > LCA > CA [66,74]. The function of the microbial-derived SBA UDCA as a ligand for FXR is determined by conjugation status [75]. Unconjugated UDCA and glycine-conjugated GUDCA act as FXR antagonists, whereas taurine-conjugated TUDCA is an FXR agonist [68,69,75]. In mice, tauro-β-muricholic acid (TβMCA) has also been shown to be an FXR antagonist [67]. Several other BA-activated nuclear receptors, namely, the vitamin D receptor [76,77,78,79,80], the pregnane X receptor (PXR) [81,82,83], and the constitutive androstane receptor (CAR) [66,84], have also been described and are summarized in Figure 3. Importantly, BAs are not the primary signaling chemicals for these receptors, and limited information is available in veterinary medicine characterizing these in the context of BA activation [66]. From human and rodent studies, FXR is expressed in intestinal epithelial cells, hepatocytes, white blood cells, including monocytes and T cells, proximal tubular cells of the kidney, within the adrenal glands, lungs, adipose tissue, and the brain [66,70,71,72,73,85]. In health, FXR signaling regulates cholesterol synthesis, host BA synthesis, and lipid metabolism through modulation of the hormone fibroblast growth factor (FGF) 15/FGF19 [85]. In particular, in the terminal ileum, FXR activation by BAs releases FGF15/FGF19 which modulates host hepatic *de novo* BA synthesis [85]. In human medicine, the role of BA-activated FXR is also well described in many disease states including intestinal inflammation, precancerous and cancerous colorectal lesions, appetite regulation, skeletal muscle mass, obesity, hepatic nutrient metabolism, and pulmonary inflammation from SARS-CoV-2 infection [66,73,85,86]. Given the myriad potential applications in human medicine, many FXR-targeted therapeutics are currently within preclinical or clinical study, including for metabolic and liver disease [87] as well as cancer [88].

The influence of BA-activated FXR is far less characterized in veterinary medicine. An important difference between dogs and humans is the expression of FXRβ as a functional receptor in dogs, whereas in humans, FXRβ is a pseudogene that is not expressed [89,90]. The functional significance of FXRβ is currently unknown [90]. In healthy beagles (*n* = 7), the hepatic expression of FXR mRNA transcripts is not influenced by dietary fat, as seen when comparing a low-fat diet (Hill’s i/d Low-Fat diet) to a diet with 20% fat [91]. In dogs with COMMD-1 gene deficiency and subsequent hepatic copper accumulation, hepatic mRNA production from genes regulated downstream by FXR was reduced, similar to Wilson’s disease in humans; however, FXR expression was not directly measured in this study [92]. These two studies constitute the extent of functional FXR characterization in dogs or cats to date. However, applying translational principles of FXR knowledge from human and rodent studies may open new avenues for therapeutic intervention in companion animals. For instance, with the recent description of bile acid diarrhea (BAD) in dogs [93,94] and the high likelihood of BAD being underrecognized in veterinary patients with diarrhea, a characterization of the FXR-FGF15/FGF19 axis in dogs may provide additional treatment considerations to modulate the BA pools. Given the importance of FXR and the paucity of information in veterinary medicine, further characterization of FXR, including FXRβ, and FXR-mediated processes in dogs and cats is warranted along with other BA-activated receptors including the vitamin D receptor, PXR, and CAR.

The other major type of host BA-activated receptors is the membrane-bound G protein-coupled receptor. The best characterized of these is TGR5 [95,96,97,98,99]. Two additional receptors in this category are α5β1 integrin [100] and sphingosine-1-phosphate receptor 2 (S1PR2) [101,102,103,104,105], which are summarized along with TGR5 in Figure 3. TGR5 has preferential binding affinity for microbial-derived SBAs with the order of affinity being LCA > DCA > CDCA > CA [66,95]. Of these, BAs conjugated with taurine have higher affinity than glycine conjugates or unconjugated BAs, and notably, UDCA also possesses the ability to serve as a TGR5 ligand [97]. Based on human and murine studies, TGR5 expression is largely ubiquitous and includes all segments of the gastrointestinal tract, visceral organs, central nervous system, lymphatics, heart, lungs, skeletal muscle, adipose, and endocrine glands [96]. Functionally, TGR5 is involved in intestinal electrolyte transport, gastrointestinal motility, the regulation of inflammation through NF-κB antagonism, glucagon-like peptide 1 (GLP-1) secretion, and human gastric adenocarcinoma biology [66,106,107,108]. This diverse biologic activity of TGR5 has led to investigations of targeted therapeutic approaches in human medicine for the treatment of obesity [109], type 2 diabetes [110], and inflammatory diseases [111]. 

Within veterinary medicine, less is known regarding TGR5, though the canine expression pattern of TGR5 has been described [99]. In healthy dogs (*n* = 8), TGR5 expression, evaluated via immunohistochemistry and RNA in situ hybridization, revealed expression in canine tissue from the tongue, esophagus, stomach, duodenum, jejunum, ileum, cecum, colon, rectum, liver, gallbladder, and pancreas [99]. Within these canine tissues, endothelial cells and macrophages (membrane and intracytoplasmic) strongly expressed TGR5 [99]. A conference abstract from Manchester et al. reported that canine macrophages (MH588) express TGR5 in vitro [112]. Further, when canine macrophages are pre-treated for two hours in culture with the SBA LCA, they produce significantly greater anti-inflammatory IL-10 and significantly less pro-inflammatory TNFα cytokines [112]. The PBA CDCA has a similar effect on TNFα cytokine responses but did not impact IL-10, and CA did not impact either response [112]. This represents the only mechanistic investigation of TGR5-mediated activity in veterinary species and appears to recapitulate the anti-inflammatory properties mediated through microbial-derived SBAs acting as a TGR5 ligand on mononuclear immune cells as described in humans [98]. To date, the expression of TGR5 in cats has not been characterized. Moreover, additional functional roles mediated through TGR5 in dogs and cats remain unexplored. Recently, Jergens et al. highlighted the potential role for dogs with diabetes mellitus as a naturally occurring model of Type 2 diabetes mellitus due to observed alterations in the canine gut microbiota and BA pools seen in this disease and implicated the role of BA signaling mediated by TGR5 and/or FXR [113].This suggestion is strengthened by the known pancreatic expression of TGR5 in dogs [99]. Given the widespread expression of canine TGR5, there are likely additional spontaneous disease parallels in the context of inflammatory diseases, obesity, and metabolic diseases where TGR5 is recognized to play a role in human disease. The continued investigation of the BA-activated TGR5 in companion animals will be impactful for identifying novel therapeutic applications to both veterinary and human medicine.

In order to fully understand the physiologic impact of collaborative BA metabolism between the gut microbiome and canine and feline hosts, further characterization of host BA-activated receptors in a canine- and feline-specific context is required. This understanding is in the early stages in companion animals, especially given the lack of characterization of FXR in dogs or cats. In veterinary medicine, more information exists describing canine and feline BA pools in health and disease, which will subsequently be discussed. 

## 3. Canine Microbial-Derived Bile Acids

Over the past 5 years, microbial-derived BAs have been increasingly studied in canines (Table 1). Studies in healthy dogs have characterized the microbial-derived BAs during the development of puppies [114], as well as the impact of antimicrobials [59,115,116,117,118] and diet [119,120,121,122,123,124,125,126] on the BA pools of healthy dogs. Additional studies on disease states have largely focused on the context of gastrointestinal [52,64,127,128,129,130,131] and pancreatic disease [128,132], with additional information characterizing microbial-derived BAs in canine obesity [119,125], diabetes mellitus [113], and myxomatous mitral valve disease (MMVD) [133]. The following sections of this review detail the existing veterinary literature describing microbial-derived BAs in dogs.

### 3.1. Canine Microbial-Derived Bile Acids in Health

In puppies, the normal development of BAs is well described [114]. As the gut microbiota is established early in life, the metabolic potential of the gut microbes to convert host-derived PBAs into SBAs also develops. This phenomenon is well documented in humans, where microbial-derived SBAs are in low abundance early in life but increase in infants by six months of age [134]. Dynamic BA pool maturation in guide dog puppies (*n* = 53) have been described from 1 week of age up to 16 weeks [114]. In these puppies, host-derived PBAs, CA and CDCA, predominated in the feces for at least the first 6 weeks of life, with the microbial-derived SBAs LCA and DCA barely detectable [114]. Interestingly, UDCA is the first microbial-derived SBA noted in puppy feces, as it significantly increases between weeks three and four of life, while only 2/13 puppy fecal samples had LCA or DCA detected by five to six weeks of age [114]. UDCA is often produced through bacterial HSDH activity (Figure 1), and applying metagenomic sequencing could help identify gut microbes with the genetic potential for this BA biotransformation in this age group of puppies. By 7–9 weeks of age, the canine fecal BA composition is dominated by microbial-derived SBAs, and this persists in dogs greater than one year old [114]. These results coincided with the increasing abundance of *P. hiranonis* (formerly *C. hiranonis*; detected via qPCR) over the same time period [114]. Given that *P. hiranonis* is known to perform 7*α*-dehydroxylation (Figure 1), which is a critical step for the formation of microbial-derived SBAs, this provides evidence for microbial community shifts directly impacting BA pool dynamics [114]. Collectively, fecal BAs pool development and maturation are described in puppies, but biogeographic resolution along the gastrointestinal tract is missing. Lastly, an asymptomatic carriage of *C. difficile* (detected via qPCR) was present in the puppy feces during the first six weeks of life [114], as has been previously reported [135]. As *P. hiranonis* becomes established in the puppy gut microbiome, SBAs are subsequently produced and colonization with *C. difficile* abates. This relationship between the presence of microbial-derived SBAs and decreased *C. difficile* colonization is well established in humans [11,114,136]. Further, the asymptomatic *C. difficile* colonization of puppies recapitulates what is seen in human infants [137]. However, it is important to note that the severity of *C. difficile* infection seen in adult human is not observed in adult dogs [138], making this a potentially important area for One Health discovery to explain the differential pathogenic activity of *C. difficile* in dogs and people. 

Although a specific study has not set out to define and characterize BA profiles in healthy dogs, we can leverage adult healthy controls included in other studies to accomplish this objective. Collectively, these studies demonstrate that the fecal BA profile in healthy dogs is compositionally 80–90% microbial-derived SBAs [59,64,113,114,117,118,121,122,123,124,127,128]. Based on 16S amplicon sequencing performed on duodenal, ileal, colonic, and rectal content from healthy dogs (*n* = 6), a subsequent analysis of identified taxa using PICRUSt (phylogenetic investigation of communities through reconstruction of unobserved states) predicted significant changes to expected BA deconjugation pathways along the length of the canine gastrointestinal tract [139]. Specifically, the BSH enzyme choloylglycine hydrolase, which deconjugates BAs, had an increased abundance of expected expression moving aborally through the intestinal tract [139]. No pathways related to the conversion of PBAs into SBAs were described in the analysis, although alpha-methylacyl-CoA racemase, which is an enzyme capable of CA or CDCA synthesis from cholesterol, was noted to have higher expected expression in the canine duodenum [139]. In these same healthy dogs, the biogeographic resolution of microbial-derived metabolites using gas chromatography–time-of-flight mass spectrometry (GC-TOF-MS) has been described, but unfortunately, the untargeted methodology employed did not detect any primary or secondary BAs, a known limitation of this technique [139]. In healthy dogs, to date, there are no multi-omics studies integrating metagenomic sequencing and targeted BA metabolomics. Therefore, in healthy dogs, additional multi-omics studies are required to characterize collaborative BA metabolism, specifically describing canine gut microbes mechanistically responsible for BA biotransformations in health. In the following sections, the impact of antimicrobials [59,115,116,117,118] and diet [119,120,121,122,123,124,125,126] on the BA pools of healthy dogs will be reviewed. 

### 3.2. Effects of Antimicrobials on Canine Microbial-Derived Bile Acids 

Antimicrobial administration in dogs, specifically Tylosin [59,118], metronidazole [115,117] and a combination of metronidazole with enrofloxacin [116], significantly alter the canine gut microbiome and subsequently impact microbial-derived BA metabolism. Specifically, following antimicrobial administration, a BA dysmetabolism, characterized by a significant reduction in microbial-derived SBAs and a significant excess of host-derived PBAs, is observed [59,115,116,117,118].

For Tylosin, even just a 7-day course leads to significant alterations in the canine gut microbiota and a sequential reduction in microbial-derived SBAs [59,118]. Specifically, during a prospective, randomized, placebo-controlled study of 16 healthy client-owned dogs given Tylosin (20 mg/kg PO q12h for 7d) or placebo, alterations to microbial community structure (assessed via 16S amplicon sequencing) and subsequent fecal BA dysmetabolism were noted immediately after Tylosin administration, which persisted up to eight weeks after [118]. *P. hiranonis* abundance (detected via qPCR) significantly decreased immediately after Tylosin administration with a concurrent rise of the host PBAs CA and CDCA, which became statistically significant compared to the placebo by day 21 and day 63 of the study [118]. In the placebo group consisting of healthy dogs, the median SBA composition at baseline was 95.2% and remained similar across three timepoints up to eight weeks (97.9%, 97.7%, and 93.4%, respectively) [118]. Although it did not reach statistical significance, likely due to inter-dog variability, dogs receiving Tylosin at baseline had BA pools predominated by microbial-derived SBAs (93.9%) which precipitously dropped to 14.5% immediately after Tylosin, 10% at 2 weeks post antimicrobial administration, and only recovered to 22.9% eight weeks post antimicrobial administration [118]. This captures how quickly a dramatic microbial dysbiosis can induce a BA dysmetabolism, characterized by a reduction in microbial-derived SBAs production, and how these microbial ecosystem alterations can persist for up to 8 weeks post antimicrobial administration. 

To address Tylosin-induced dysbiosis and subsequent BA dysmetabolism, a prospective, randomized, placebo-controlled study evaluated the potential benefit of fecal microbiota transplantation (FMT) to mitigate these microbial ecosystem effects induced by antimicrobial administration [59]. In this study, 16 healthy purpose-bred research dogs given Tylosin (20 mg/kg PO q24h for 7d) received either a single FMT enema (10 mL/kg) on day 9 (*n* = 6), oral FMT capsules (two capsules PO q24h) from days 8–21 (*n* = 6), or placebo capsules from days 8–21 (*n* = 10) [59]. In agreement with the prior study conducted by Manchester et al. [118], seven days of Tylosin administration induced dysbiosis (based on the qPCR canine dysbiosis index), characterized by significantly reduced *P. hiranonis*, and fecal BA dysmetabolism, characterized by decreased microbial-derived SBAs in all dogs [59]. However, in this study, regardless of treatment, by day 14, the canine dysbiosis index normalized and fecal SBAs predominated for all dogs, except in two dogs that did not receive FMT [59]. In those two dogs, *P. hiranonis* was still significantly decreased, and a concurrent BA dysmetabolism characterized by a reduction in microbial-derived SBAs persisted [59]. Ultimately, this study did not statistically find a benefit for FMT compared to the placebo for accelerating recovery from Tylosin-induced dysbiosis and subsequent BA dysmetabolism [59]. A key difference between both studies evaluating Tylosin-induced dysbiosis and subsequent BA dysmetabolism is the study populations utilized. Persistent disturbances in microbial BA metabolism were demonstrated in client-owned dogs [118], whereas in purpose-breed research dogs, regardless of receiving FMT or not, the microbial community dynamics were more resistant to tylosin perturbation which could have been influenced by previous use in other experiments [59]. These studies highlight the importance of differential microbiome responses based on the population of dogs evaluated, which needs to be considered when designing studies with microbiome outcomes.

Metronidazole also induces dysbiosis and subsequently reduces microbial-derived BAs, leading to BA dysmetabolism [115,117]. A prospective, nonrandomized study of 24 healthy client-owned dogs evaluated the effects of metronidazole (15 mg/kg PO q12h for 14d) on the gut microbiota and fecal BA pools [117]. This study consisted of three groups: a control group (*n* = 8) maintained on their historic diet and which did not receive metronidazole, a group (*n* = 8) first transitioned to a hydrolyzed soy diet for six weeks and then given metronidazole, and a third group (*n* = 8) maintained on their historic diet and given metronidazole [117]. Fecal samples were collected after the first and second weeks post metronidazole administration [117]. Regardless of diet, dogs receiving metronidazole demonstrated a persistent elevation in the canine dysbiosis index including decreased *P. hiranonis*, even 4 weeks post antimicrobial administration [117]. This coincided with a BA dysmetabolism characterized by a significant decreased in microbial-derived SBAs, specifically LCA and DCA, and a significant increase in host-derived PBAs, specifically CA and CDCA, compared to pre-antimicrobial administration [117]. The SBAs DCA and LCA remained significantly decreased from baseline two weeks after the cessation of metronidazole, with LCA still significantly decreased from baseline at 4 weeks post metronidazole administration [117]. The reduction in microbial-derived SBAs significantly correlated with a decreased abundance of *P. hiranonis* (detected via qPCR) [117]. 

Recently, the impact of a prebiotic to abate metronidazole-induced dysbiosis and subsequent BA dysmetabolism has been evaluated in healthy dogs (*n* = 12) [115]. In this 8-week crossover study, all dogs received a baseline diet for two weeks, then metronidazole (20 mg/kg PO q12h for 14d) was administered to all dogs, followed by a subset of dogs receiving a prebiotic (novel biosimilar milk oligosaccharides) added to their diet for six weeks, while the remaining dogs did not [115]. Then, dogs not receiving the prebiotic were administered it, resulting in all dogs receiving both treatments. As previously demonstrated [117], metronidazole induced a dysbiosis defined by decreased microbial diversity and alterations in the relative abundance of 20 bacterial genera and families (assessed via 16S amplicon sequencing) with a concurrent reduction in microbial-derived SBAs [115]. Dogs who received the prebiotic demonstrated a faster recovery of microbial-derived SBAs, specifically DCA and UDCA, compared to dogs that did not receive the prebiotic [115]. Although *P. hiranonis* is considered the primary BA converter in dogs, in this study, there were no differences in *P. hiranonis* (detected by qPCR) between the treatment groups, despite the normalization of BA metabolism in dogs given a prebiotic post metronidazole administration [115]. This suggests that *P. hiranonis* may not be the only member of the canine microbiota responsible for SBA generation via the *bai* operon. Additionally, since post metronidazole prebiotic administration quickened the recovery of microbial-derived SBAs without a change in *P. hiranonis* abundance, this could also be related to BSH activity by other gut microbes and/or the increased metabolic capability of *P. hiranonis*, and further multi-omics studies are needed to further elucidate the underlying mechanism. 

Lastly, the combination of metronidazole and enrofloxacin has also been demonstrated to induce dysbiosis and a sequential reduction in microbial-derived BAs. In a prospective, randomized, placebo-controlled double-blinded study of 22 healthy research dogs given both enrofloxacin (10 mg/kg PO q24h for 21d) and metronidazole (12.5 mg/kg PO q12h for 21d), the mitigation effects of a concurrent probiotic and synbiotic administration were assessed [116]. The probiotic component was Proviable^®^ which was designed to contain 1 × 10^10^ colony-forming units (CFUs) of a proprietary mixture of *Bifidobacterium bifidum*, *Enterococcus faecium*, *Streptococcus thermophilus*, *Lactobacillus acidophilus*, *L. bulgaricus*, *L. casei*, and *L. plantarum* [116]. The synbiotic component was Mycequin^®^ which was designed to contain 1 × 10^10^ CFU of a proprietary strain of *Saccharomyces boulardii* and the prebiotic beta-glucan [116]. After a one-week baseline period, a subset of healthy dogs (*n* = 11) received the probiotic/synbiotic combination one hour following the administration of antimicrobials, and the remaining dogs (*n* = 11) did not [116]. After an eight-week washout period, this study was completed in a crossover design such that all dogs received the prebiotic/symbiotic combination treatment post antimicrobials [116]. The broad-spectrum antimicrobial combination, metronidazole and enrofloxacin, induced a dysbiosis with reduced microbial diversity (assessed with 16S amplicon sequencing), including a significant decrease in *P. hiranonis* (detected via qPCR), and sequential BA dysmetabolism [116]. The concurrent BA dysmetabolism occurred with a significant reduction in microbial-derived SBAs, specifically LCA and DCA, with a concurrent increase in host-derived PBAs, specifically CA compared to pre-antimicrobials [116]. This antimicrobial-induced dysbiosis and BA dysmetabolism occurred regardless of prebiotic/synbiotic administration [116]. Eight weeks post antimicrobial administration, the SBA DCA normalized in dogs receiving the prebiotic/synbiotic combination but remained significantly decreased in dogs that did not [116]. This indicates that the prebiotic/synbiotic combination may provide dogs with a recovery and/or resilience benefit allowing the gut microbial community to regain BA metabolism function, including BSH activity and 7*α*-dehydroxylation activity, following a broad-spectrum antimicrobial administration. 

Collectively, in healthy dogs, antimicrobials induce dysbiosis, often including a significant decrease in *P. hiranonis*, a key microbe in BA metabolism, resulting in sequential BA dysmetabolism, characterized by decreased microbial-derived SBAs. To date, mitigation strategies, such as FMT, probiotics, prebiotics, and synbiotics, to correct antimicrobial-induced dysbiosis and subsequent BA dysmetabolism are promising, but limited evidence is available, thus highlighting an important area of potential discovery in veterinary microbiome medicine.

### 3.3. Impact of Diet on Canine Microbial-Derived Bile Acids 

The impact of diet composition on both the gut microbiota and microbial-derived BAs has been evaluated in healthy dogs. As expected, diet impacts the gut microbiota and microbial-derived SBAs, but the response is dependent on the diet fed. Diet types studied to date in dogs include the following: high protein, high fat, low fiber (HP-HF-LF) [126]; high protein, high fat, low fiber consistent with bones and raw food (BARF) [121]; high protein, high fiber (HP-HF) [122]; and grain-free diets [120,123,124].

A HP-HF-LF diet has been shown to increase the fecal microbial-derived SBA DCA [126]. Specifically, client-owned healthy dogs (*n* = 8) were fed a commercial kibble diet (Felleskjøpet’s Labb adult [140]) for two weeks, and then boiled minced beef was added in increasing amounts over the next three weeks, resulting in dogs consuming 5% of their total energy requirement from minced beef [126,140]. All dogs returned to eating the commercial kibble diet alone for an additional two weeks, making the total study duration seven weeks [126]. When dogs were consuming the 75% minced beef, a HP-HF-LF diet, significant alterations in the fecal microbial population and reduced microbial diversity (assessed via 16S amplicon sequencing) was observed compared to dogs consuming only the kibble diet [140]. Specifically, dogs fed minced beef had a higher abundance of a microbe of the Clostridiaceae family with 97% of its identity shared with *P. hiranonis* [140]. Additionally, these microbiome alterations occur simultaneously with a significant increase in the fecal microbial-derived SBA DCA [126]. When minced beef was removed from the diet and dogs only consumed the commercial kibble diet, a significant decrease in the fecal microbial-derived SBA UDCA and taurine-conjugated BAs occurs [126]. Further investigation is needed to determine mechanistically why feeding dogs minced beef consistent with a HP-HF-LF diet results in alterations in the microbial community that promotes *P. hiranonis* and the microbial production of DCA. 

Regarding BARF diets, consistent with high protein, high fat, and low fiber, there are no differences in fecal BAs or the abundance of *P. hiranonis* reported [121]. In a study of 46 client-owned healthy dogs, whether they were fed a BARF diet (*n* = 27) or a commercial diet (*n* = 19), alterations in fecal microbiota (assessed via 16S amplicon sequencing) were noted between the diets, but no differences in the abundance of *P. hiranonis* (detected via qPCR) were reported [121]. It is important to note that both the BARF and commercial diets were not standardized. The BARF diets contained many different animal-sourced proteins, with a mean diet composition of 44.4% crude protein, 28.4% crude fat, and 2.69% crude fiber on a dry matter basis [121]. The commercial diets that were fed contained significantly less crude protein and crude fat and significantly more crude fiber than the BARF diets [121]. Interestingly, no differences in fecal primary or secondary BAs were found between the diet groups [121]. It is unclear from this manuscript if an untargeted metabolomics (GC-TOF-MS) versus targeted metabolomics approach was utilized. Regardless, given that BAs were presented as total primary BAs and total secondary BAs, it was not possible to corroborate if DCA was also specifically enriched by feeding healthy dogs BARF diets, as was seen with feeding healthy dogs boiled minced beef [126]. 

When dogs were fed a high-protein and high-fiber diet, no alterations in fecal microbial-derived BAs were reported [122]. Healthy female research dogs (*n* = 28) were fed a control diet for a period of five weeks with 22.3% crude protein and 12.1% total dietary fiber on a dry matter basis [122]. Four dogs had a sham spay surgery performed on them and were continued on the control diet for 24 weeks [122]. The remaining dogs all underwent spay surgery and then were continued on either the control diet (*n* = 8), a high-protein and high-fiber diet with 42.0% crude protein and 20.9% total dietary fiber on a dry matter basis (*n* = 8), or the high-protein and high-fiber diet supplemented with omega-3 and medium-chain fatty acids (*n* = 8) for 24 weeks [122]. The fecal microbial community structure (assessed via 16S amplicon sequencing) differed between dogs fed the control diet and those fed the high-protein and high-fiber diet, with omega-3 and medium-chain fatty acid supplementation having minimal impact [122]. Specifically, the genera *Faecalibacterium*, *Rombustia*, and *Fusobacterium* increased in abundance, and *Catenibacterium* decreased in abundance in dogs fed the high-protein and high-fiber diet [122]. Among all spayed dogs, diet did not impact fecal microbial-derived BAs, though the PBA CA was high in dogs fed the control diet [122]. Among the sham-operated dogs and spayed dogs both fed the control diet, there were also no significant changes to fecal microbial-derived SBAs, though the PBA CDCA was increased in the sham-operated dogs compared to dogs who were spayed [122]. In total, in this study, there were no significant differences in fecal microbial-derived BAs when dogs transitioned to a high-protein and high-fiber diet. 

In three studies on dogs fed grain-free diets, minimal impact on fecal microbial-derived BAs is described [120,123,124]. In the first study, healthy research beagle dogs (*n* = 12) were fed a standard laboratory diet for a period of two weeks and then were transitioned to either a grain-based (*n* = 6) or grain-free (*n* = 6) diet for an additional four weeks [124]. Dogs fed a grain-free diet had a significant increase in fecal host-derived PBA CA after four weeks; however, there were no differences in either microbial-derived BA measures, specifically DCA and LCA [124]. In the second study, healthy labrador retrievers (*n* = 8) were fed a grain-free, commercially available diet for 26 weeks [123]. After 26 weeks, dogs fed a grain-free diet had significantly greater concentrations of fecal PBAs CA and CDCA as well as the microbial-derived SBA DCA [123]. However, these alterations in BAs, did not result in a significant change in the overall BA pool composition [123]. At baseline, the fecal BA pool was predominately SBAs (98.5%) within only 1.5% PBAs, and after 26 weeks of feeding a grain-free diet, the BA profiles were similar at 97.1% SBAs and 2.9% PBAs [123]. In the last study, healthy research beagle dogs (*n* = 8) were fed one of four grain-free fava bean-based diets in a 2 × 2 Latin square design that compared the fermentation status of the fava bean flour in the diet and the tannin content in the diet (specifically, unfermented low tannin, fermented low tannin, unfermented high tannin, and fermented high tannin) [120]. Using an unvalidated colorimetric assay to detect total fecal BA, the only difference detected was between both low tannin diets, where the fermented fava bean diet had significantly decreased total BAs [120]. Overall, these three studies demonstrated alterations in fecal BAs following feeding a grain-free diet to healthy dogs. Given the lack of multi-omic studies in dogs fed grain-free diets, it is difficult to ascertain the mechanism for the observed changes in microbial-derived BAs, and thus it is unclear if the fecal BA alterations are biologically significant.

Collectively, the impact of diet on microbial-derived SBAs in healthy dogs appears to be minimal, with the one exception of increased fecal DCA in dogs fed a HP-HF-LF diet of boiled minced beef that corresponded with an increased abundance of *P. hiranonis* [126,140]. This minimal dietary impact contrasts with the dramatic impact of antimicrobials on microbial-derived SBAs. Likely, this difference is attributed to antimicrobials inducing a significant dysbiosis resulting in the elimination of microbes essential and required for BA metabolism. It is important to note that dietary changes alter nutrient availability and microbial niches inducing a dysbiosis [141,142], but this dysbiotic state likely does not eliminate the microbes responsible for BA metabolism, thus preserving BA pool composition. 

### 3.4. Impact of Gastrointestinal and Pancreatic Diseases on Canine Microbial-Derived Bile Acids 

Dogs with gastrointestinal diseases, including canine chronic inflammatory enteropathy (CIE) [52,64,127,128,130,131], exocrine pancreatic insufficiency (EPI) [128], and canine non-infectious acute diarrhea [129], have alterations in BA pools consistent with BA dysmetabolism. Herein, evidence for each disease state will be presented and reviewed. 

The disease process of canine CIE has been recently reviewed [143], but briefly, it is characterized by gastrointestinal signs lasting at least three weeks that result from a complex pathogenesis of host inflammatory dysregulation, genetics, and environmental factors that include alterations in the gut microbiota, not unlike human IBD. Classically, CIE is categorized based on therapeutic responses, specifically, food-responsive enteropathy (FRE), immunosuppressant-responsive enteropathy (IRE) or steroid-responsive enteropathy (SRE), and non-responsive enteropathy (NRE). Historically, a fourth category of patients who responded to an empiric course of antimicrobial therapy has been termed antibiotic-responsive enteropathy (ARE); however, the appropriateness of empiric antimicrobial therapy in canine CIE is currently debated, with this subgroup of patients proposed to be renamed “microbiome-responsive enteropathy (MRE)”, as these CIE dogs appear to clinically benefit from treatments targeting the gut microbiome, including, but not limited to, fiber supplementation, probiotics, and FMT [143]. 

To date, the most robust multi-omics study in canine CIE found that a 14-day dietary therapeutic trial with a hydrolyzed soy diet resulted in the abatement of dysbiosis leading to the production of microbial-derived SABs through a *P. hiranonis* mechanism [52]. This study applied both 16S amplicon and metagenomic sequencing to characterize the microbial population of CIE dogs (*n* = 29) paired with a targeted assessment of 15 BAs via ultra-performance liquid chromatography (UPLC). Follow-up in vitro culture assays and bacterial isolate whole genome sequencing were employed to mechanistically explore why dogs in the study clinically improved on this hydrolyzed diet trial [52]. Specifically, patients included in the study had histologic confirmation of intestinal inflammation and were excluded if they had been treated with a hydrolyzed protein diet, antibiotics, corticosteroids, or probiotics in the previous two weeks [52]. Once enrolled, dietary therapy was attempted with a hydrolyzed protein diet for 14 days, and if remission occurred, patients were considered diet responsive (*n* = 20) [52]. If remission did not occur, patients were termed non-diet responsive (*n* = 9) and were treated with metronidazole (10 mg/kg PO q12h) for 14 days while remaining on the hydrolyzed diet [52]. If remission was still not achieved, prednisone (1 mg/kg PO q12h) was then added for the final 14 days [52]. Diet-responsive and non-diet-responsive patients were compared, and metagenomic data revealed that the relative abundance of *P. hiranonis* significantly increased in the diet-responsive CIE dogs, corresponding with a significant increase in the microbial-derived SBAs DCA and LCA [52]. From 16S amplicon sequencing, it was seen that non-diet-responsive patients had an increased relative abundance of *E. coli* and *Clostridium perfringens*, two bacterial species associated with promoting CIE [52]. Isolates of both these bacteria obtained from patients with CIE in the study were cultured in vitro, and physiologically relevant concentrations of DCA added to the culture inhibited the growth of both bacteria, though *C. perfringens* was more significantly inhibited than *E. coli* [52]. The inclusion of this culture experiment highlights that microbial-derived SBAs modulate the microbial community and can limit the growth of potentially deleterious community members [52]. To better characterize the microbial mechanisms of BA conversion, fecal metagenomics was performed for the presence of both BSH genes capable of performing deconjugation and the *bai* operon for microbes capable of performing 7*α*-dehydroxylation required to produce SBAs like DCA and LCA [52]. In total, 31 bacteria belonging to the genera *Lactobacillus*, *Streptococcus*, and *Eubacterium* contained BSH genes [52]. Through correlation analysis, several of these bacteria genera were positively correlated with SBA concentrations [52], highlighting the importance of the deconjugation step of PBAs, encoded by the BSH gene, to allow further BA biotransformations in dogs. Separately, two isolates of *P. hiranonis* obtained from the feces of diet-responsive patients with CIE were submitted for whole genome sequencing, and an intact *bai* operon was identified, confirming the mechanistic capability of a canine isolate performing 7*α*-dehydroxylation [52]. In total, this multi-omics study highlights the mechanistic importance of microbial-derived SBAs in canine CIE, with diet-responsive patients recovering the *bai* operon function in *P. hiranonis*. Meanwhile, non-diet-responsive patients were unable to recover microbial-derived SBAs, warranting further investigation to identify therapeutic interventions that may promote these mechanisms, particularly in dogs suffering from NRE. 

Three additional studies [64,127,128] included in a recent meta-analysis [144] corroborate BA dysmetabolism characterized by an increase in host-derived PBAs and a decrease in microbial-derived SBAs as a biomarker for CIE in dogs. When compared to healthy dogs (*n* = 11), dogs suffering from canine CIE (*n* = 24) demonstrated significant dysbiosis [64], as assessed via the previously validated qPCR canine dysbiosis index [53]. BA dysmetabolism, characterized by a significant decrease in total microbial-derived SBAs, specifically DCA, and a concurrent increase in the percentage of host-derived PBA CDCA were reported [64]. Comparing SRE dogs (*n* = 23; 0.5–1 mg/kg PO q12h x 3wks and then tapered in an unstandardized manner) to healthy controls (*n* = 24) retrospectively for up to 12 weeks, significant dysbiosis based on an increased canine dysbiosis index and a significant decrease in *P. hiranonis*, as well as in *Faecalibacterium* and *Fusobacterium*, were observed [127]. At baseline, SRE dogs had significant BA dysmetabolism characterized by a decrease in microbial-derived SBAs DCA, LCA, and UDCA and an increase in the host-derived PBA CA [127]. At one month and then two to three months post steroid administration, a significant recovery of microbial-derived SBAs LCA and DCA was noted, which coincided with a significant increase in *P. hiranonis* and a significant clinical improvement based on the validated canine inflammatory bowel disease activity index (CIBDAI) [127]. Importantly, this study describes BA dysmetabolism in SRE and captures that steroid treatment promotes the restoration of microbial-derived SBAs which coincides with clinical improvement, similar to what was shown by Wang et al. in FRE over a 2-week duration [52,127].

Importantly, not all dogs suffering from CIE have a significant reduction in *P. hiranonis* abundance, despite displaying BA dysmetabolism. For example, in a recent study comparing CIE dogs (*n* = 15) to healthy dogs (*n* = 34), the canine dysbiosis index revealed significant dysbiosis with a reduction in *Faecalibacterium* abundance, but *P. hiranonis* abundance was not significantly different between the groups, despite significant reductions in the fecal microbial-derived SBAs DCA and LCA in this population of CIE dogs [128]. Although these findings are not fully understood, this could indicate diminished BSH activity thus preventing BA deconjugation, the critical first step in microbial BA metabolism. Further studies are required to elucidate the underlying mechanism of this discrepancy in some CIE dogs that have *P. hiranonis* present but lack microbial-derived SBAs.

It is well established that some Yorkshire terriers with CIE have concurrent panhypoproteinemia or discrete hypoalbuminemia, a clinical syndrome known as protein-losing enteropathy, which can be managed with a low-fat dietary intervention [145]. Compared to healthy Yorkshire terriers (*n* = 26), Yorkshire terriers with CIE ± PLE have dysbiosis, based on the canine dysbiosis index, with a significant reduction in *P. hiranonis* and *Fusobacterium* abundance [130]. Bile acid dysmetabolism was also observed, with a significant reduction in microbial-derived SBA UDCA but no significant alteration in DCA or LCA compared to healthy Yorkshire terriers [130]. In a cohort of Yorkshire terriers (*n* = 11) suffering from CIE and followed up to clinical remission, unexpectedly, a significantly lower percentage of SBAs was seen than in those with active CIE [130]. The mean percentage of SBAs in CIE Yorkshire terriers in clinical remission was only 22% (range: 2–73%), compared to 50% (range: 4–100%) during active CIE in Yorkshire terriers and 78% (range: 5–99%) in healthy Yorkshire terriers [130]. This is the only study documenting BA dysmetabolism with decreased microbial-derived SBAs in CIE dogs in clinical remission compared to active CIE. Of note, all Yorkshire terriers, despite a healthy status or disease state, demonstrated a wide range in fecal BA composition, which warrants further investigation to determine if either microbial, host-related, or breed-related mechanism(s) can explain high inter-dog variability in the fecal BA profiles observed. 

Lastly, in CIE dogs (*n* = 18) with a low CCECAI [146], consistent with clinically insignificant disease, dysbiosis (based on the qPCR canine dysbiosis index) and BA dysmetabolism were not observed prior to treatment [131]. These CIE dogs were then fed a standardized home-cooked diet (crude protein 39.2%, crude fat 7.9%, crude fiber 1.3% on a dry matter basis). Not surprisingly, following dietary intervention, dysbiosis and BA derangements still were not observed [131]. This is likely attributed to the minimally impacted CIE dog population utilized in this study. 

Based on the current available literature, there is strong evidence for BA dysmetabolism, characterized by a reduction in microbial-derived SBAs, during active canine CIE. Notably, the recovery of microbial-derived BAs coincides with clinical improvement, regardless of the therapeutic intervention required to achieve clinical remission. Further investigation into discrepancies in this trend, as seen in Yorkshire terriers with CIE ± PLE [130] and CIE dogs with a low CCECAI [131], warrant multi-omics studies to distill mechanism. Additionally, multi-omics studies could be utilized to determine microbiome signatures and BA profiles that could predict responses to therapeutics in dogs suffering from CIE.

Dogs with EPI have BA dysmetabolism characterized by a reduction in microbial-derived SBAs [128,132]. Compared to healthy dogs (*n* = 34), dogs with EPI treated with pancreatic enzymes (*n* = 29), or ones that are even untreated (*n* = 7), demonstrate fecal dysbiosis (based on the qPCR canine dysbiosis index) with a significant reduction in *P. hiranonis* and *Fusobacterium* along with significantly increased *E. coli* [128]. Fecal dysbiosis corresponds with a significant reduction in the microbial-derived SBAs DCA and LCA in EPI dogs, regardless of treatment status, and a significant reduction in UDCA in treated EPI dogs compared to healthy dogs [128]. These findings are corroborated in another study which reported a significant reduction in total fecal SBAs in EPI dogs (*n* = 20) compared to healthy dogs (*n* = 10) [132]. In this study, a reduction in fecal SBAs was significantly inversely correlated with zonulin, a marker of mucosal barrier integrity [132]. Zonulin was also significantly inversely correlated with the serum of taurohyodeoxycholic acid (THCA), a conjugated microbial-derived SBA [132]. In summary, this supports the notion that EPI is associated with BA dysmetabolism characterized by decreased microbial-derived SBAs. Additionally, these findings warrant a broader exploration of the role SBAs may play in the regulation of the mucosal barrier function and inflammation in dogs. 

Bile acid dysmetabolism characterized by a reduction in microbial-derived SBAs is reported during episodes of canine non-infectious acute diarrhea (NAD). Specifically, dogs with NAD (*n* = 18) have fecal dysbiosis (based on the qPCR canine dysbiosis index) with a significant decrease in *P. hiranonis* and *Faecalibacterium* and an increase in *E. coli* abundance [129]. Additionally, via 16S amplicon sequencing, compared to healthy dogs, dogs with NAD have reduced microbial diversity and alterations in microbial community structure, consistent with dysbiosis which coincided with BA dysmetabolism characterized by a significant decrease in microbial-derived SBA LCA and a significant increase in PBA CA [129]. Dogs with NAD treated with FMT (5 g/kg rectal enema in saline; *n* = 11) displayed an improvement in microbial diversity by day 7 post FMT [129] compared to NAD dogs receiving metronidazole (15 mg/kg PO q12h for 7; *n* = 7), which, at day 7, experienced a further reduction in microbial diversity [129]. Furthermore, the microbial community structure was significantly different at day 7 between NAD dogs treated with FMT compared to those that received metronidazole [129]. A recovery of *P. hiranonis* was noted by day 7 in dogs receiving FMT, while an abundance of *P. hiranonis* was further reduced in NAD dogs prescribed metronidazole [129]. In NAD dogs who received FMT, the BA dysmetabolism (decreased LCA; increased CA) noted at baseline had normalized by day 7 and remained normalized at day 28, while NAD dogs who received metronidazole had a worsened BA dysmetabolism, with even less LCA at day 7 and day 28 [129]. Additionally, significant improvement in the fecal score in FMT-treated dogs was observed at day 7, which was significantly improved compared to the metronidazole-treated dogs, even at day 28 [129]. This study describes BA dysmetabolism in canine NAD and documents the correction of BA dysmetabolism through the use of FMT which provided superior clinical resolution compared to an antimicrobial which further exacerbated the BA dysmetabolism.

In summary, there are commonalities across the gastrointestinal and pancreatic diseases studied in the context of microbial-derived BAs in dogs. These data demonstrate that CIE, EPI, and canine NAD are associated with BA dysmetabolism where microbial-derived SBAs are reduced during active disease. For CIE and NAD, there is additional evidence that the improvement of active disease corresponds with the restoration of fecal microbial-derived SBAs, which more closely represented BA pools observed in healthy dogs. In the future, additional attention to therapies that promote the restoration of microbial-derived SBA pools is warranted in canine gastrointestinal and pancreatic diseases. It is also notable that currently there are no published reports describing microbial-mediated BA metabolism in canine hepatic disease. Hepatic disease has the potential to alter BA metabolism through host and microbial mechanisms, which likely impacts the interplay of the host and intestinal microbiota via the host-gut microbiota–BA axis. Characterizing this dynamic may open new insights into therapeutic interventions for dogs suffering from hepatic diseases, as it has in human medicine [147,148,149]. 

### 3.5. Impact of Non-Gastrointestinal Disease on Canine Microbial-Derived Bile Acids 

Beyond gastrointestinal and pancreatic diseases in dogs, there have been several other disease states where microbial-derived BAs have been characterized and a BA dysmetabolism exists. To date, these disease states include: obesity [119,125], diabetes mellitus [113], and myxomatous mitral valve disease (MMVD) [133].

Although a significant BA dysmetabolism has not been documented in obese canines, shifts in BA pools are described in overweight and obese dogs who lose weight [119,125]. In one study, overweight and obese research Beagle dogs (*n* = 12, mean BCS 7.9 ± 0.75) fed a high-protein, high-fiber diet (crude protein 42.04%, total dietary fiber 26.81% on a dry matter basis) were assessed over 24-week weight loss period [119]. During weight loss, significant alterations in microbial community structure (assessed via 16S amplicon sequencing) were noted [119]. Specifically, weight loss corresponded with a significant increase in the relative abundances of *Proteobacteria*, *Bifidobacterium*, *Coriobacteriaceae* UCG-002, undefined *Muribaculaceae*, *Allobaculum*, *Eubacterium*, *Negativibacillus*, *Ruminococcus gauvreauii* group, uncultured *Erysipelotrichaceae*, and *Parasutterella* [119]. Conversely, weight loss lead to a significant reduction in the relative abundances of *Prevotellaceae* Ga6A1 group, *Catenibacterium*, *Erysipelatoclostridium*, *Holdemanella*, *Lachnoclostridium*, *Lactobacillus*, *Megamonas*, *Peptoclostridium*, *Ruminococcus gnavus* group, and *Streptococcus* [119]. The fecal BA composition in overweight and obese dogs was predominated by microbial-derived SBAs (95%) with only 5% PBAs [119]. However, of the microbial-derived SBAs measured, UDCA significantly increased by 12 weeks of weight loss and DCA significantly decreased by 24 weeks [119]. These shifts in fecal BA pools corresponded with reduction in the inflammatory markers IL-6 and c-reactive protein at week 24 following weight loss [119]. This study highlights that alterations in microbial populations occur during weight loss in tandem with shifts in the BA pools and reduced systemic inflammation in obese dogs undergoing weight loss. 

From a different study, overweight and obese research Beagle dogs (*n* = 9, mean BCS 7.8) were fed a high-protein, high-fiber diet (crude protein 38.6%, total dietary fiber 7.2% on dry matter basis) to maintain their overweight phenotype for the duration of study [125]. Using a 3 × 3 Latin square design, all dogs received the inulin prebiotic Orafti^®^ SIPX at a low dose of 1 g/d PO, a high dose of 2 g/d PO, or a placebo capsule of cellulose [125]. Each treatment was given for 14 days, then followed by a 14 day washout period before the next treatment was started [125]. No significant differences in microbial diversity or microbial community structure (assessed via 16S amplicon sequencing) were noted between treatments [125]. In the high dose prebiotic treatment group, *Turicibacter* (detected via qPCR) was significantly greater [125]. No significant differences were detected in fecal BA concentrations between treatment groups, though microbial-derived SBAs DCA and LCA tended to increase in the high dose prebiotic group compared to the low dose (*p* = 0.08 and 0.09, respectively) [125]. From these data, it is suggested that use of an inulin prebiotic may promote microbial production of SBAs in overweight and obese dogs; however, additional study within a larger population is likely required to confirm this finding and determine the clinical and physiologic benefit.

Though not as dramatic as in gastrointestinal and pancreatic diseases, shifts in microbial BA metabolism occur in canine obesity, including promoting UDCA production during weight loss [119]. The rise in the microbial-derived SBA UDCA corresponded with significant reduction in the inflammatory markers IL-6 and c-reactive protein [119]. Obesity is known to promote both a dysbiosis and BA dysmetabolism in people and rodent models which corresponds with increased systemic inflammation [150]. Though obesity-related inflammation is multifactorial in nature, there are anti-inflammatory properties described for UDCA, including downregulation of IL-6 mRNA and protein expression in macrophages [151]. It is possible that microbial-derived UDCA is a mechanism that contributes to resolution of obesity-related inflammation in dogs, and thus warrants further exploration.

Recently, BA dysmetabolism has been demonstrated in dogs with diabetes mellitus. Compared to healthy dogs (*n* = 10), dogs with diabetes mellitus (*n* = 10) had enrichment in the fecal relative abundance of *Enterobacteriaceae* without overt alteration of microbial diversity or microbial community structure (assessed via 16S amplicon sequencing) [113]. Dogs with diabetes mellitus had a BA dysmetabolism characterized by a significant decreased in the microbial-derived SBA LCA and a significant increase in host-derived PBA CA compared to healthy dogs [113]. Separately, a study of untargeted serum metabolomic profiles comparing dogs with diabetes mellitus (*n* = 6) and healthy controls (*n* = 6) identified decreased TCDCA, TDCA, and TUDCA in the serum of diabetic dogs, though no microbiota or fecal BA characterization were assessed in this study [152]. Though still early in discovery, BA dysmetabolism in canine diabetes mellitus may provide an additional target for therapeutic intervention in the future. In humans there is strong evidence for the connection between BA dysmetabolism and type 2 diabetes mellitus, prompting current investigation of several bile acid-centered therapeutic strategies for this disease [153]. Moreover, the commonly utilized pharmaceutical metformin, originally thought to reduce hyperglycemia through direct effects on gluconeogenesis in the liver, has been demonstrated also to alter gut microbiota and increase the conjugated SBA GUDCA which in turn regulates glucose metabolism through FXR signaling [68]. Considering the BA dysmetabolism described in dogs with diabetes mellitus is similar to what is seen in humans, dogs may serve as a translational animal model for human disease. Likewise, emergence of BA therapeutics for diabetic patients may be a future reality within veterinary medicine as they continue to be explored for human diabetic patients in a translational medicine context.

Within the context of heart disease, BA dysmetabolism is documented in dogs with MMVD. Compared to healthy controls (*n* = 17), dogs with MVVD (total *n* = 75: Stage B1, *n* = 23; Stage B2, *n* = 27; Stage C or D, *n* = 25) have reduced microbial diversity and distinct microbial community structure (assessed via 16S amplicon sequencing) [133]. However, there is no difference in microbial community structure between stages of MMVD [133]. Dogs with severe MMVD (Stage C/D) displayed a significant dysbiosis (based on qPCR canine dysbiosis index) compared to healthy dogs [133]. Interestingly, these dogs with severe MMVD had a decreased abundance of *P. hiranonis* (detected by qPCR) [133]. The only difference in fecal BAs detected in this study was significantly greater GCA in MMVD Stage B2 dogs compared to healthy and MMVD Stage B1 dogs; however, GCA did not differ from stage C or D dogs [133]. Evidence from human and rodent studies implicate BA dysmetabolism in a variety of heart disease, including GCA being shown to induce arrhythmic atrial contractions in a dose-dependent manner [154,155]. So, while a stronger link between dysbiosis and MMVD was demonstrated in the canine study, including a trending reduction in *P. hiranonis*, there is evidence of a BA dysmetabolism characterized by an increase in host-derived PBA GCA. Taken together with the reduction in *P. hiranonis*, it is possible that reduced capacity of the gut microbiota to perform BA biotransformations leads to the observed increase in GCA in MMVD dogs. Further studies could investigate if this phenomenon is present in dogs with arrhythmias, as has been explored in humans.

**Table 1:** Summary of published peer-reviewed literature on canine microbial-derived bile acids. Evidence-based medicine levels are as follows: 1, high-quality randomized trial; 2, lesser-quality randomized trial or prospective comparative study; 3, case-control study or retrospective comparative study; 4, case series; and 5, expert opinion. Abbreviations: CE = chronic enteropathy, CIE = chronic inflammatory enteropathy, DI = dysbiosis index qPCR panel, DM = diabetes mellitus, EPI = exocrine pancreatic insufficiency, GC-MS = gas chromatography and mass spectrometry, GC-TOF-MS = gas chromatography–time-of-flight mass spectrometry, GI = gastrointestinal, HC = healthy control, HPLC = high performance liquid chromatography, HSDH = hydroxysteroid dehydrogenase, LC-MS/MS = liquid chromatography–tandem mass spectrometry, MMVD = myxomatous mitral valve disease, NAD = noninfectious acute diarrhea, OW = overweight, PBA = primary bile acid, SBA = secondary bile acid, SRE = steroid responsive chronic enteropathy, URI = upper respiratory infection, UPLC = ultra performance liquid chromatography. Primary bile acid abbreviations used: CA = cholic acid, CDCA = chenodeoxycholic acid, GCA = glycocholic acid, GCDCA = glycochenodeoxycholic acid, TCA = taurocholic acid, TCDCA = taurochenodeoxycholic acid, αMCA = alpha muricholic acid, βMCA = beta muricholic acid. Secondary bile acid abbreviations used: DCA = deoxycholic acid, ECA = epicholic acid, GDCA = glycodeoxycholic acid, GLCA = glycolithocholic acid, GUDCA = glycoursodeoxycholic acid, HCA = hyocholic acid, HDCA = hyodeoxycholic acid, LCA = lithocholic acid, TDCA = taurodeoxycholic acid, TLCA = taurolithocholic acid, TUDCA = tauroursodeoxycholic acid, UCA = ursocholic acid, UDCA = ursodeoxycholic acid, γMCA = gamma muricholic acid, ωMCA = omega muricholic acid.

Author and Year	Study Context	Disease Status	Bile Acid Sample	Primary Bile Acids Reported	Secondary Bile Acids Reported	Bile Acid Assessment Method	Microbiota Assessment Method	Evidence Level
Honneffer et al., 2017 [139]	Healthy	HC (*n* = 6)	3h post-mortem intestinal contents	None	None	GC-TOF-MS, PICRUSt	16S rRNA amplicon	4
Blake et al., 2020 [114]	Development	HC (*n* = 86)	Lyophilized feces	CA, CDCA	DCA, LCA, UDCA	GC-MS	DI, enteric pathogen qPCR	2
Belchik et al., 2023 [115]	Antimicrobials, diet	HC (*n* = 12)	Lyophilized feces	CA, CDCA	DCA, LCA, UDCA	GC-MS	DI, 16S rRNA amplicon	2
Marclay et al., 2022 [59]	Antimicrobials, FMT	HC (*n* = 16)	Lyophilized feces	CA, CDCA	DCA, LCA, UDCA	GC-MS	DI	1
Whittemore et al., 2021 [116]	Antimicrobials	HC (*n* = 22)	Lyophilized feces	CA	DCA, LCA	GC-TOF-MS	DI, 16S rRNA amplicon	1
Pilla et al., 2020 [117]	Antimicrobials	HC (*n* = 24)	Lyophilized feces	CA, CDCA	DCA, LCA	GC-MS	DI, 16S rRNA amplicon	2
Manchester et al., 2019 [118]	Antimicrobials	HC (*n* = 16)	Lyophilized feces	CA, CDCA	DCA, LCA, UDCA	GC-MS	DI, 16S rRNA amplicon	1
Phungviwatnikul et al., 2021 [122]	Diet	HC (*n* = 28)	Lyophilized feces	CA, CDCA	DCA, LCA, UDCA	GC-MS	16S rRNA amplicon	2
Reis et al., 2021 [120]	Diet	HC (*n* = 8)	Voided feces	N/A	N/A	Colorimetric Total BA	None	2
Donadelli et al., 2020 [123]	Diet	HC (*n* = 8)	Lyophilized feces	CA, CDCA	DCA, LCA, UDCA	GC-MS	None	4
Pezzali et al., 2020 [124]	Diet	HC (*n* = 12)	Fresh frozen feces	CA, CDCA	DCA, LCA	HPLC	None	2
Schmidt et al., 2018 [121]	Diet	No GI Disease (*n* = 46)	Lyophilized feces	All primary together	All secondary together	GC-TOF-MS	DI, 16S rRNA amplicon	2
Herstad et al., 2018 [126]	Diet	HC (*n* = 8)	Freeze-dried feces	CA, CDCA, GCA, GCDCA, TCA. TCDCA	DCA, GDCA, GLCA, GUDCA, LCA, TLCA, TDCA, TUDCA, UDCA	LC-MS/MS	16S rRNA amplicon in prior publication	4
Vecchiato et al., 2023 [131]	CIE	CIE (*n* = 18)	Lyophilized feces	CA, CDCA	DCA, LCA, UDCA	GC-MS	DI	4
Galler et al., 2022 [130]	CIE	HC (*n* = 26), CIE (*n* = 14)	Lyophilized feces	CA, CDCA	DCA, LCA, UDCA	GC-MS	DI	3
Blake et al., 2019 [128]	CIE, EPI	HC (*n* = 34), CIE (*n* = 15), EPI (*n* = 36)	Lyophilized feces	CA, CDCA	DCA, LCA, UDCA	GC-MS	DI, 16S rRNA amplicon	3
Guard et al., 2019 [127]	SRE	HC (*n* = 24), SRE (*n* = 23)	Lyophilized feces	CA, CDCA	DCA, LCA, UDCA	GC-MS	DI	3
Wang et al., 2019 [52]	CIE	HC (*n* = 24), CIE (*n* = 29)	Voided feces	CA, CDCA, GCA, GCDCA, TCA, TCDCA, αMCA, βMCA	DCA, GDCA, LCA, TDCA, TLCA, γMCA, ωMCA	UPLC	Metagenomics, 16S rRNA amplicon	2
Giaretta et al., 2018 [64]	CIE	HC (*n* = 11), CIE (*n* = 24)	Lyophilized feces	CA, CDCA	DCA, LCA, UDCA	GC-MS	DI	3
Chaitman et al., 2020 [129]	NAD	HC (*n* = 14), NAD (*n* = 18)	Lyophilized feces	CA, CDCA	DCA, LCA UDCA	GC-MS	DI, 16S rRNA amplicon	3
Phungviwatnikul et al., 2022 [119]	Diet, Overweight	OW (*n* = 12)	Lyophilized feces	CA, CDCA	DCA, LCA UDCA	GC-MS	16S rRNA amplicon	2
Alexander et al., 2018 [125]	Diet, overweight	OW (*n* = 9)	Lyophilized feces	CA	3-oxoCDCA, 7-oxoDCA, DCA, isoLCA, LCA	HPLC	DI, 16S rRNA amplicon	2
Li et al., 2021 [133]	MMVD	HC (*n* = 17), MMVD (*n* = 75)	Voided feces	CA, CDCA, GCA, GCDCA, TCA, TCDCA	DCA, GDCA, GLCA, GUDCA, LCA, TDCA, TLCA, TUDCA, UDCA	LC-MS/MS, UPLC	DI, 16S rRNA amplicon	3
Jergens et al., 2019 [113]	DM	HC (*n* = 10), DM (*n* = 10)	Lyophilized feces	CA, CDCA	DCA, LCA, UDCA	GC-MS	16S rRNA amplicon	3

### 3.6. Conclusions Regarding Canine Microbial-Derived Bile Acids

Within the past five years, the investigation into microbial-derived SBAs in dogs has rapidly expanded. It is evident that in healthy canines, the fecal BA pools are dominated by microbial-derived SBAs. To date, the largest drivers of BA dysmetabolism are those that induce dysbiosis, such as antimicrobials, gastrointestinal disease, and pancreatic disease. Based on the current evidence, dietary modifications can also impact BA pools, though typically microbial-derived SBAs are preserved. Extra-intestinal diseases, such as obesity, diabetes mellitus, and MMVD, have been minimally explored, but also each is accompanied by a disease-specific BA dysmetabolism. Importantly, no studies have evaluated the impact of canine hepatic disease on microbial BA metabolism, thus, highlighting an area of future investigation in canine medicine. 

Most canine studies reviewed herein have utilized qPCR or 16S amplicon sequencing to draw conclusions about shifts in the gut microbial community structure, with only one study utilizing metagenomic sequencing [52]. Furthermore, most studies have employed targeted BA metabolomics to specifically evaluate only a limited subset of unconjugated bile acids, which may limit our comprehensive understanding of disease-specific BA dysmetabolism, thus diminishing our discovery of bile acid-directed therapeutic interventions in veterinary medicine. When a broader range of BAs are evaluated, including conjugated BAs, additional understandings of canine disease-specific BA dysmetabolism and therapeutic potentials may be elucidated. To date, only three studies have employed targeted metabolomics approaches to capture conjugated BAs [52,126,133]. Additionally, novel microbial conjugated BAs have not yet been described in dogs, which likely contribute to host physiology via BA-activated receptors. By leveraging multi-omics approaches, specifically studies that pair metagenomics with expanded targeted BA metabolomics, will continue to expand our knowledge on states of BA dysmetabolism in dogs with the ultimate goal of identifying potential BA-directed therapeutic targets. Continued work to characterize microbial-derived BAs in this manner will provide a mechanistic understanding of the host-gut microbiome–BA axis in health and states of disease aimed at improving the healthspan of dogs.

## 4. Feline Microbial-Derived Bile Acids

Over the past 5 years, microbial-derived BAs have also been increasingly studied in cats (Table 2). However, compared to dogs, limited information is available. For example, unlike in dogs, no studies depict the normal development of BA pools in healthy cats. Presently, studies are largely limited to evaluations of healthy cats within the context of diet modifications [156,157,158] and antimicrobial administration [159,160], or in states of disease such as kittens with upper respiratory infections (URIs) [161], feline CKD [162], and feline chronic enteropathy [163]. Herein, the current evidence available regarding feline BAs will be reviewed.

### 4.1. Feline Microbial-Derived Bile Acids in Health 

Currently, there is a paucity of information regarding BA development from kittenhood to adulthood. Therefore, this section is focused on BAs in healthy adult cats; specifically, the impact of dietary modifications and antimicrobial administration on feline microbial-derived SBAs. Diet composition drives alterations in the gut microbiota [164] and thus has the potential to modify microbial-derived BA pools. 

In healthy research cats (*n* = 10), alterations in fecal BAs were observed based on the formulation of diet (wet vs. dry) and macronutrient profiles [158]. Cats fed the dry formulation had a three-fold decrease in total fecal BAs compared to cats fed the wet formulation [158]. In this study, in addition to the difference in moisture content, the wet food diet had approximately four times the amount of fat and half the amount of total fiber on a dry matter basis, compared to the dry diet [158]. This finding implies that higher fat and lower fiber diets induce an increase in total fecal BAs in cats. More recently, two identically formulated dry food diets that varied only in the extrusion preparation method were compared in healthy research cats (*n* = 36) [157]. The extrusion preparation method impacts the amount of digestion-resistant starch present in the diet, which can serve as a nutrient source for members of the gut microbiota. Cats in the study either received a diet with low digestion-resistant starch (*n* = 17) or high digestion-resistant starch (*n* = 19) [157]. Cats fed the high digestion-resistant starch diet had significantly greater microbial diversity, and their microbial community structures (assessed via 16S amplicon sequencing) were significantly different from cats fed the low digestion-resistant starch diet [157]. Genera enriched in the high digestion-resistant starch group included *Lactobacillus*, *Eubacterium*, *Odoribacter*, *Treponema*, *Stenotrophomonas*, and *Peptococcus* [157]. Of the 23 fecal BAs measured, 12 increased in cats fed the high digestion-resistant starch diet and only 3 decreased relative to cats fed the low resistant-starch diet [157]. The largest magnitude of changes occurred with the PBAs cholate and taurocholenate sulfate and the SBAs 12-dehydrocholate and 3-dehydrocholate [157]. Given that most feline PBAs are conjugated and not sulfated, both PBAs reported here are likely still the product of microbial biotransformations. Thus, even when the macronutrient composition of diets are equivalent, diet preparation techniques, which influence the availability of macronutrients to the gut microbiota, impact microbial BA metabolism.

Additional evidence for changes in microbial-derived BAs in response to diet composition is available in elderly healthy research cats (*n* = 40) between the ages of 8.3 and 13.5 years [156]. All cats received a geriatric-formulated diet compared to a traditional control diet for 30 days in a crossover study design that included a 30-day washout period between study periods. Cats fed the geriatric-formulated diet displayed an increase in the relative abundance of *Coriobacteriaceae*, *Veillonellaceae*, *Bifidobacteriaceae*, and *Lactobacillaceae* (assessed via 16S amplicon sequencing) [156]. Subsequently, a significant decrease in the fecal microbial-derived SBAs DCA, LCA, dehydro-LCA, UDCA, iso-UDCA, and 7a-hydroxycholestenone with a concurrent increase in the fecal PBA CA were observed [156]. Furthermore, the relative abundance of *P. hiranonis* negatively correlated with CA and positively correlated with all observed SBAs, as would be expected given this microbe’s known 7*α*-dehydroxylation ability [156]. Notably, other microbes had stronger correlations, including a microbe from the family *Coriobacteriaceae*, genus *Adlercreutzia*, which had the strongest negative correlation with CA and strongest positive correlation with dehydro-LCA, DCA, and LCA as well as a positive correlation with iso-UDCA [156]. Interestingly, microbes from the family *Coriobacteriaceae*, including those from the genus *Adlercreutzia*, have been demonstrated to contain an NAD(P)H-dependent oxidoreductase with 12a-HSDH activity, which confers the ability to alter the 12th carbon position of CA in the conversion to UDCA [165]. Other microbes found to positively correlate with SBAs belonged to the genera *Mogibacterium*, *Coprococcus*, *Paraprevotella*, and *Salmonella* [156]. These correlations may exist as a result of other concurrent metabolic processes. In the case of *Adlercreutzia*, it is also known to produce the isoflavan equol, which was in the elderly cats fed a standard feline diet [156]. Collectively, diet composition impacts the feline gut microbiota and thus subsequentially alters the feline BA pools. Based on the limited literature on cats, specific microbiome alterations induced by diet are impossible to predict. 

The other context in which fecal microbial-derived BAs have been evaluated in healthy cats is post antimicrobial administration, specifically clindamycin [159,160]. In the first study, healthy adult research cats that received clindamycin (75 mg PO q24h for 21d) with either a placebo (*n* = 8) or an oral synbiotic (*n* = 8; 5 billion CFUs of *B. bifidum*, *E. faecium*, *S. thermophilus*, *L. acidophilus*, *L. bulgaricus*, *L. casei*, and *L. lantarum* in a proprietary mixture along with a proprietary blend of fructooligosaccharide and arabinogalactan) [159]. Antimicrobial administration induced significant dysbiosis characterized by a reduction in microbial diversity and a significant alteration in the microbial community structure which was persistent for up to 630 days (assessed via 16S amplicon sequencing) [159]. Additionally, the feline dysbiosis index was significantly elevated post-antimicrobial treatment in all cats; however, the cats receiving the synbiotic displayed a significantly worse feline dysbiosis index compared to cats receiving the placebo [159]. Following antimicrobial treatment, the microbial-derived SBA DCA was significantly reduced in all cats [159]. Additionally, by day 630, post antimicrobial administration, DCA concentrations normalized to baseline levels [159]. Notably, this study utilized an untargeted metabolomic approach, so the only microbial-derived SBA detected was DCA [159]. From this study on healthy cats, clindamycin induced significant dysbiosis and subsequent BA dysmetabolism characterized by a decreased production of the microbial-derived SBA DCA with minimal improvement with symbiotic administration [159]. 

In a similar study, healthy adult research cats received a higher clindamycin dose (150 mg PO q24h for 21d) in tandem with either a placebo (*n* = 8) or the same oral synbiotic as the previous study (*n* = 8) and were followed up to 6 weeks post antimicrobial administration [160]. Regardless of the placebo or symbiotic treatment, a significant reduction in microbial diversity and altered microbial community structure (assessed via 16S amplicon sequencing) was detected immediately post antimicrobial treatment and persisted for six weeks [160]. In alignment with these findings, the feline dysbiosis index demonstrated significant dysbiosis post antimicrobial treatment which persisted at 6 weeks post antimicrobial treatment [160]. Immediately following antimicrobial treatment, microbial-derived SBA DCA was significantly reduced and remained significantly decreased at 6 weeks post antimicrobials. It is important to note that symbiotic treatment did not abate the gut microbiome and/or BA dysmetabolism post antimicrobial treatment [160]. 

Collectively, in healthy cats, the antimicrobial clindamycin induces dysbiosis and subsequent BA dysmetabolism characterized by a decrease in microbial-derived SBA DCA. Derangements to the microbial ecosystem can persist up to six weeks following the discontinuation of clindamycin; however, the feline microbiome can recover, and BA dysmetabolism is no longer present by 1.5 years post antimicrobial administration [159]. Crucially, the co-administration of a symbiotic does not abate the antimicrobial-induced alterations or subsequent BA dysmetabolism [159,160]. 

### 4.2. Feline Microbial-Derived Bile Acids in Disease

Microbial-derived SBAs have also been studied in states of disease in cats including kittens with URIs given antimicrobials [161], feline CKD [162,166], and feline CE [163]. The development and maturation of the fecal BA pools has recently been characterized in kittens with URIs starting at 2 months of age up to approximately one year [161]. URI-inflicted kittens either received amoxicillin/clavulanic acid (20 mg/kg PO q12h for 20d; *n* = 15), doxycycline (10 mg/kg PO q24h for 28d; *n* = 15), or did not receive any antimicrobials (*n* = 15) [161]. Fecal BAs were assessed serially at day 0, upon completion of antimicrobial therapy (day 20 for amoxicillin/clavulanic acid and day 28 for doxycycline and control groups), and day 60, 120, and 300 [161]. The microbiota of these kittens were assessed using 16S amplicon sequencing and the feline dysbiosis index in a separate publication [167]. At the end of the antimicrobial treatment, kittens receiving amoxicillin/clavulanic acid had significantly reduced microbial diversity compared to both kittens that received doxycycline or no antimicrobials, but this was not significantly different from its own baseline [167]. Given that the gut microbial population dynamically matures in complexity over the first year of life in cats [168], it is difficult to know if these microbiome alterations in kittens administered amoxicillin/clavulanic acid can be interpreted as abnormal [167]. Immediately post antimicrobials, with either amoxicillin/clavulanic acid or doxycycline, a significant alteration in the gut microbial community structure was observed compared to kittens not receiving antimicrobials [167]. In URI-inflicted kittens not receiving antimicrobials, microbial-derived SBAs DCA and LCA increased serially and, by day 300, were significantly increased compared to the baseline [167]. By comparison, at the conclusion of amoxicillin/clavulanic acid and doxycycline treatments, there was a significant decrease in DCA and LCA [167]. By day 60, DCA and LCA did not differ between either antimicrobial-treated URI kittens or those untreated [167]. Importantly, *P. hiranonis* (detected via qPCR) abundance did not significantly differ in URI kittens given antimicrobials or not, perhaps due to inter-individual variation or possibly indicating other factors beyond *P. hiranonis* impacting the production of microbial-derived SBAs in cats [167]. It is important to also interpret this study in light of the antimicrobials administered during concurrent URI and not to attribute findings as caused singularly by URI or a particular antimicrobial. This study lends further evidence for antimicrobial-induced dysbiosis and subsequent BA dysmetabolism, characterized by decreased microbial-derived DCA and LCA, and provides a glimpse into longitudinal BA maturation in kittens suffering from URI early in life. 

Bile acid dysmetabolism is also observed in cats suffering from CKD. Compared to healthy cats (*n* = 10), cats with CKD (total *n* = 10: IRIS Stage 1, *n* = 5; Stage 2, *n* = 4; Stage 3, *n* = 1), being fed the same complete and balanced dry renal therapeutic diet for 14 days, demonstrated no significant difference in fecal BAs, despite a robust profile of BAs being assessed [166]. These cats were then fed diets that varied in fiber sources for 4 weeks, with one diet containing short-chain fructooligosaccharides and one containing a more soluble fiber source of apple pomace [166]. Again, no differences were noted in fecal BA profiles between healthy and CKD cats in their responses to the varied fiber sources [166]. However, the fiber source did significantly impact the feline fecal BA profile, resulting in significantly more TCDCA, TUDCA, and hyocholate detected in cats fed the apple pomace fiber source [166]. Despite these findings, a recent abstract from Summers et al. describes fecal BA dysmetabolism in cats with CKD [162]. Fecal BAs of healthy senior cats (*n* = 10) were compared to CKD cats (total *n* = 29: IRIS Stage 2, *n* = 17; IRIS Stage 3 or 4, *n* = 12) [162]. The microbial-derived SBA UDCA was significantly decreased in cats with CKD and had a weak but significant negative correlation with creatinine [162]. Given that the nuclear receptor FXR is known to be expressed in the kidney and can modulate inflammation, it is possible that the BA dysmetabolism described by Summers et al. could promote CKD progression by losing the anti-inflammatory action of UDCA when it acts as an FXR ligand within the kidney (Table 1). From the two reports presented, there is evidence of BA dysmetabolism in feline CKD; however, it has not been robustly characterized or mechanistically explored and is currently limited by a lack of multi-omics studies including simultaneous assessments of the gut microbiota.

Currently, there is minimal information related to BA metabolism in feline gastrointestinal disease. Recently, dysbiosis (assessed via 16S amplicon sequencing and the qPCR feline dysbiosis index) has been described in feline chronic enteropathy compared to healthy cats [54]. Though paired fecal BA data are not available, this study did find that cats with CE had a significant decrease in *P. hiranonis* (detected via qPCR) which was below the reference interval for healthy cats in 35% (24/68) of CE cats [54].

Separately, the characterization of differential fecal metabolites in cats with IBD and intestinal small cell lymphoma was also recently performed [169]. Interestingly, of the 84 differential metabolites identified, none were BAs [169]. That study utilized lyophilized homogenized fecal samples and an untargeted metabolomic approach which is not optimized to detect BAs [169]. It is important to note that upstream sample handling can impact metabolite concentrations, and the process of lyophilization prior to targeted fecal BA metabolomics in humans was recently shown to increase BA concentrations by about two- to four-fold compared to non-lyophilized feces when then assessed via LC-MS/MS [170]. This thus highlights the importance of consistent methods of performing targeted metabolomics and the importance of understanding the employed methods. 

In a recent conference abstract from Chi-Hsuan Sung et al., fecal BAs in feline CE, comparing cats with IBD (*n* = 22) or intestinal small cell lymphoma (*n* = 34) to healthy cats, (*n* = 45) were described [163]. Cats with CE had increased total fecal BAs compared to healthy cats [163]. In this study, 14% (8/56) of CE cats had an increase in host-derived PBAs, and 23% (13/56) of CE cats had an abnormal primary to secondary BA profile [163]. *P. hiranonis* abundance (detected with qPCR) revealed a significant negative correlation with total PBAs [163], as would be expected given its 7*α*-dehydroxylation capability. There were no reported differences between cats with IBD or intestinal small cell lymphoma [163]. So, while less robustly characterized than dogs, cats with CE do appear to experience dysbiosis and subsequent BA dysmetabolism characterized by a loss of microbial-derived SBAs and an abundance of host-derived PBAs. Further research leveraging multi-omics data sets are needed to elucidate the underlying mechanisms leading to BA dysmetabolism being observed.

**Table 2:** Summary of published peer-reviewed literature on feline microbial-derived bile acids. Evidence-based medicine levels are as follows: 1, high-quality randomized trial; 2, lesser-quality randomized trial or prospective comparative study; 3, case-control study or retrospective comparative study; 4, case series; and 5, expert opinion. * Sulfated bile acids also assessed. Abbreviations: CKD = chronic kidney disease, DI = dysbiosis index qPCR panel, GC-MS = gas chromatography and mass spectrometry, GC-TOF-MS = gas chromatography–time-of-flight mass spectrometry, HC = healthy control, HPLC = high performance liquid chromatography, LC-MS/MS = liquid chromatography–tandem mass spectrometry, URI = upper respiratory infection, UPLC = ultra performance liquid chromatography. Primary bile acid abbreviations used: CA = cholic acid, CDCA = chenodeoxycholic acid, GCA = glycocholic acid, GCDCA = glycochenodeoxycholic acid, TCA = taurocholic acid, TCDCA = taurochenodeoxycholic acid, αMCA = alpha muricholic acid, βMCA = beta muricholic acid. Secondary bile acid abbreviations used: DCA = deoxycholic acid, ECA = epicholic acid, GDCA = glycodeoxycholic acid, GLCA = glycolithocholic acid, GUDCA = glycoursodeoxycholic acid, HCA = hyocholic acid, HDCA = hyodeoxycholic acid, LCA = lithocholic acid, TDCA = taurodeoxycholic acid, TLCA = taurolithocholic acid, TUDCA = tauroursodeoxycholic acid, UCA = ursocholic acid, UDCA = ursodeoxycholic acid, γMCA = gamma muricholic acid, ωMCA = omega muricholic acid.

Author and Year	Study Context	Disease Status	Bile Acid Sample	Primary Bile Acids Reported	Secondary Bile Acids Reported	Bile Acid Assessment Method	Microbiota Assessment Method	Evidence Level
Ephraim and Jewell, 2021 [156]	Diet, aging	HC (*n* = 40)	Frozen feces homogenate	CA	7α-hydroxy cholestenone, dehydroLCA, DCA, isoUDCA, LCA, UDCA	GC-MS, LC-MS	16S rRNA Amplicon	2
Jackson et al., 2020 [157]	Diet	HC (*n* = 36)	Frozen feces homogenate	* CA, CDCA, GCDCA, TCA, TCDCA	* 3-dehydroCA, 6-oxoLCA, 7-ketoDCA, 7-ketoLCA, 12-dehydroCA, dehydroLCA, DCA, GDCA, GLCA, HCA, isoHDCA, isoUDCA, LCA, TDCA, UCA	GC-MS, LC-MS	16S rRNA Amplicon	2
Anantharaman-Barr et al., 1994 [158]	Diet	HC (*n* = 10)	Lyophilized feces	CA, CDCA	DCA, LCA, UDCA + HDCA	GC-MS	None	4
Whittemore et al., 2019 [160]	Antimicrobials	HC (*n* = 16)	Lyophilized feces	None	DCA	GC-TOF-MS	DI, 16S rRNA Amplicon	1
Whittemore et al., 2018 [159]	Antimicrobials	HC (*n* = 16)	Lyophilized feces	CA	DCA	GC-TOF-MS	DI, 16S rRNA Amplicon	1
Stavroulaki et al., 2022 [161]	Antimicrobials, development	URI (*n* = 45)	Lyophilized feces	CA, CDCA	DCA, LCA, UDCA	GC-MS	None	3
Hall et al., 2020 [166]	CKD, diet	HC (*n* = 10), CKD (*n* = 10)	Frozen feces homogenate	* CA, CDCA, GCA, TCA, TCDCA, βMCA	* 3-dehydroCA, 3β-hydroxy-5-cholenoic acid, 7-ketoDCA, 7-ketoLCA, 7α-hydroxycholestenone, 7, 12-diketoLCA, 12-dehydroCA, dehydroLCA, DCA, HCA, isoUDCA, LCA, TDCA, TLCA, TUDCA, UCA, UDCA	GC-MS, LC-MS	None	3

### 4.3. Conclusions Regarding Feline Microbial-Derived Bile Acids

Similar to dogs, within the past 5 years, there has been tremendous progress made in describing microbial-derived BAs in cats. In healthy cats, dietary composition impacts the BA pools, and importantly, antimicrobial administration, namely clindamycin [159,160], induces dysbiosis and subsequent BA dysmetabolism, specifically reducing the microbial-derived SBA DCA. Regarding kittens with diseases, kittens with URI treated with antimicrobials were shown to have transient dysbiosis and BA dysmetabolism that resolved after one month and then followed the same trajectory of BA pool diversification as age-matched kittens who did not receive antimicrobials [161,167]. Moreover, cats with CKD [162,166] and CE [163] have also been shown to experience BA dysmetabolism, though in both cases, there remains a lack of published information utilizing multi-omics approaches, pairing microbiome and targeted BA metabolomics, and data to distil mechanism(s) of observed microbial ecosystem derangements in cats. 

Lastly, where there are existing data for BA dysmetabolism in dogs, namely obesity, diabetes, and heart disease, the characterization for cats is lacking. All these diseases are prevalent in cats and thus warrant further investigation into the host-gut microbiome–BA axis. 

## 5. Overall Conclusions and Future Directions

Over the last five years, the characterization of microbial-derived BAs in companion animals is rapidly expanding. A general theme is that alterations in the gut microbiota, termed dysbiosis, in certain circumstances, leads to subsequent BA dysmetabolism, which may contribute to disease pathogenesis, thus impacting companion animal health. Now that dysbiosis-induced BA dysmetabolism is documented, following antimicrobial administration and in a variety of diseases in dogs and cats, this may represent a potential microbiome therapeutic target. Specifically, there is the potential to leverage the modulation of BA pools as a novel therapeutic intervention in disease states that typically induce BA dysmetabolism. To accomplish this in veterinary medicine, there is a need to build on the existing findings to mechanistically understand the collaborative metabolism of BAs between companion animal hosts and gut microbes in health and disease. 

Future veterinary studies should employ mechanistic study designs utilizing multi-omics approaches. Pairing metagenomic sequencing with robust targeted BA metabolomics, including the exploration of taurine- and glycine-conjugated BAs and newly described microbial amino acid-conjugated BAs, will add additional depth not currently available in the companion animal literature. Layering and integrating the gut microbiome and microbial-derived BA data with metatranscriptomic, metaproteomic, and patient outcome data will capture the mechanistic layers driving biologic outcomes. Multi-omics approaches will become the gold standard to transition microbiome science in veterinary medicine away from characterization and association toward mechanistic biologic networks of causes and effects, with the ultimate goal of discovering novel microbiome therapeutic targets to promote the extension of healthspan in companion animals.

## Data Availability

No new data were created.

## Abbreviations

BA = bile acid, *bai* = bile acid-inducible operon, BSH = bile salt hydrolase, CKD = chronic kidney disease, CE = chronic enteropathy, CIE = chronic inflammatory enteropathy, DI = dysbiosis index qPCR panel, DM = diabetes mellitus, EPI = exocrine pancreatic insufficiency, FMT = fecal microbiota transplant, GC-MS = gas chromatography and mass spectrometry, GC-TOF-MS = gas chromatography–time-of-flight mass spectrometry, GI = gastrointestinal, HC = healthy control, HPLC = high performance liquid chromatography, HSDH = hydroxysteroid dehydrogenase, LC-MS/MS = liquid chromatography–tandem mass spectrometry, MMVD = myxomatous mitral valve disease, NAD = noninfectious acute diarrhea, OW = overweight, PBA = primary bile acid, SBA = secondary bile acid, SRE = steroid responsive chronic enteropathy, URI = upper respiratory infection, UPLC = ultra performance liquid chromatography. *Primary bile acid abbreviations used:* CA = cholic acid, CDCA = chenodeoxycholic acid, GCA = glycocholic acid, GCDCA = glycochenodeoxycholic acid, TCA = taurocholic acid, TCDCA = taurochenodeoxycholic acid, αMCA = alpha muricholic acid, βMCA = beta muricholic acid. *Secondary bile acid abbreviations used:* DCA = deoxycholic acid, ECA = epicholic acid, GDCA = glycodeoxycholic acid, GLCA = glycolithocholic acid, GUDCA = glycoursodeoxycholic acid, HCA = hyocholic acid, HDCA = hyodeoxycholic acid, LCA = lithocholic acid, TDCA = taurodeoxycholic acid, TLCA = taurolithocholic acid, TUDCA = tauroursodeoxycholic acid, UCA = ursocholic acid, UDCA = ursodeoxycholic acid, γMCA = gamma muricholic acid, ωMCA = omega muricholic acid. *Amino acid abbreviations used:* Ala = alanine, Lys = lysine, Ser = serine, Thr = threonine, Leu = leucine, Ile = isoleucine, His = histidine, Phe = phenylalanine, Arg = arginine, Tyr = tyrosine, Trp = tryptophan, Met = methionine.
